# Comparison of Cytotoxicity and Antioxidant, Antibacterial, and Anti-Inflammatory Activity of Aqueous and Ethanolic Extracts from *Malus domestica*, *Prunus armeniaca*, and *Prunus cerasus* Leaves

**DOI:** 10.3390/molecules30102085

**Published:** 2025-05-08

**Authors:** Martyna Zagórska-Dziok, Aleksandra Ziemlewska, Magdalena Wójciak, Ireneusz Sowa, Ewa Wąsik-Szczepanek, Zofia Nizioł-Łukaszewska

**Affiliations:** 1 Department of Technology of Cosmetic and Pharmaceutical Products, Medical College, University of Information Technology and Management in Rzeszow, Sucharskiego 2, 35-225 Rzeszow, Poland; mzagorska@wsiz.edu.pl (M.Z.-D.); aziemlewska@wsiz.edu.pl (A.Z.); 2 Department of Analytical Chemistry, Medical University of Lublin, Aleje Raclawickie 1, 20-059 Lublin, Poland; magdalena.wojciak@umlub.pl (M.W.); ireneusz.sowa@umlub.pl (I.S.); 3 Chair and Department of Hematooncology and Bone Marrow Transplantation, Medical University of Lublin, Staszica 11, 20-081 Lublin, Poland; ewa.wasik-szczepanek@umlub.pl

**Keywords:** *Malus domestica*, *Prunus armeniaca*, *Prunus cerasus*, skin cells, antioxidants, antibacterial activity, anti-inflammatory activity, cytotoxicity

## Abstract

This study presents a comprehensive evaluation of the biological properties of aqueous and aqueous–ethanolic leaf extracts from *Malus domestica*, *Prunus armeniaca*, and *Prunus cerasus*, which are plant waste materials. Phytochemical profiles were analyzed using HPLC, and antioxidant potential was assessed via DPPH, ABTS, FRAP, and superoxide dismutase (SOD) activity assays. Extracts showed concentration-, plant-, and extract type-dependent radical scavenging effects exceeding 80%, significant Fe^3+^ ion reduction, and up to 40% enhancement of SOD activity. In vitro studies on HDF and HaCaT cells revealed reduced intracellular ROS levels, indicating antioxidant potential. Cytotoxicity assays (Alamar Blue, Neutral Red) demonstrated increased skin cell viability by up to 35% at ≤50 or 125 µg/mL, while higher doses reduced viability by up to 70%, depending on the extract. Antibacterial activity varied among plant species and was effective against seven bacterial strains. ELISA assays showed anti-inflammatory effects, with IL-1β and IL-6 levels reduced by 48% and 40%, respectively, and IL-10 increased by up to 27%. These findings suggest that the tested leaf extracts support both enzymatic and non-enzymatic antioxidant defense mechanisms and may be valuable as functional ingredients in dermatological or cosmetic applications.

## 1. Introduction

In the context of increasing environmental awareness and society’s pursuit of sustainable development, growing attention is being paid to the potential use of plant-based waste materials as valuable ingredients in cosmetic and dermatological formulations. Utilizing such raw materials not only contributes to waste reduction and environmental protection but can also provide innovative and effective active ingredients that meet the rising consumer expectations for natural and eco-friendly cosmetic products. Modern cosmetology is rapidly evolving toward the search for innovative, natural active ingredients that can serve as alternatives to synthetic substances used in cosmetic formulations. The fruit industry generates huge amounts of leaves every year, which, despite the growing interest in biomass processing technologies and the search for possible areas of use of agricultural waste, are still a raw material whose potential is insufficiently used [1]. One of the promising research directions is the use of plant-based waste, including fruit tree leaves, which have not yet found widespread application in the cosmetics industry. In the context of increasing environmental awareness and the growing popularity of the “zero waste” movement, the reuse of these raw materials aligns with the principles of sustainable development and waste minimization [2].

In this study, the leaves of *Malus domestica*, *Prunus armeniaca*, and *Prunus cerasus* were selected due to their wide availability in Central and Eastern Europe, including Poland, where these species are extensively cultivated. As a result, large amounts of leaf biomass are generated annually and typically discarded, despite their potential as a rich source of bioactive compounds. Previous research has shown that these leaves contain significant levels of polyphenols, flavonoids, and phenolic acids with promising antioxidant, anti-inflammatory, and antimicrobial properties [3,4,5,6]. However, their potential applications in skin-related therapeutic formulations remain underexplored.

Biologically active compounds in apple leaves include chlorogenic acid, ferulic acid, neochlorogenic acid, p-coumaric acid, cryptochlorogenic acid, cinnamic acid, hydroxycinnamic acid, phloridzin, phloridzin, flavanols, dihydrochalcones, quercetin, procyanidins, anthocyanins, and quercetin glycosides [3,7,8,9]. Moreover, studies show that apple leaves contain significantly more different biologically active compounds than the fruit, which may contribute to their extremely broad biological activity, also in the context of the effect on the skin [10]. The compounds present in apricot leaves are mainly chlorogenic acid, rutin, caffeoylquinic acid, coumaroyl-quinic acid, caffeoyl-glucoside, feruloylquinic acid, feruloyl-glucoside, quercetin-3-O-rutinoside, quercetin-3-O-galactoside, and kaempferol-3-O-rutinoside [5,11]. Phytochemicals contained in cherry leaves are primarily chlorogenic acid, rutin, epigallocatechin, hyperoside, isoquercitrin, avicularin, quercitrin, procyanidin B1, procyanidin B2, procyanidin C, quercetin glucoside, kaempferol glucoside, and dihydrochalcone phloridzin [4,12,13]. The broad spectrum of action of compounds contained in the extracts of the leaves of the three above-mentioned plants makes them an extremely promising raw material that can be used to eliminate a variety of skin lesions and diseases.

The aim of this work is to compare the phytochemical profile and biological activity of aqueous and ethanolic extracts from the leaves of the following three fruit trees: *Malus domestica*, *Prunus armeniaca*, and *Prunus cerasus*. This work evaluates their antioxidant, antibacterial, and anti-inflammatory properties. In order to assess the possible use of the tested raw materials in the cosmetic industry, their cytotoxicity towards skin cells in vitro and the possibility of reducing oxidative stress after exposing these cells to a pro-oxidant factor are also determined.

## 2. Results and Discussion

### 2.1. HPLC Analysis

Representative BPC (Base Peak Chromatogram) chromatograms obtained from the separation of extracts from *P. armeniaca*, *P. cerasus*, and *M. domestica* are presented in Figure S1. The corresponding MS data and qualitative analysis results are summarized in Tables S1–S3. The compounds were tentatively identified based on spectral and MS data, as well as by comparison with available literature data [14,15,16].

From each peak recorded on the chromatogram, UV-Vis spectra were collected in the range of 200–600 nm, and MS spectra were acquired in the range of 100–1200 *m*/*z*. Compounds from the flavonoid class exhibited two characteristic absorption maxima at 252–256 nm and 350–355 nm. Chlorogenic and ferulic acid derivatives showed strong absorption at 320–326 nm, while p-coumaric acid derivatives exhibited absorption at 303–306 nm. Chemical formulas were established based on MS data using MassHunter software ver 10.1. The differences between theoretical and detected masses did not exceed 5 ppm. Fragment ions were considered to confirm compound identities.

In cases where reference standards were available, they were additionally used to confirm the identity of the compounds, enhancing the reliability of the identification. In general, water–ethanol extracts from all investigated leaves were more abundant in phenolic components than water extracts, with the exception of protocatechuic acid, whose content was almost two times higher in the water extract. The predominant constituents of apricot extracts were chlorogenic acids, with a total concentration of 1302 µg/mL in PAEE and 812 µg/mL in the water–ethanol extract (PAEE), followed by quercetin 3-O-rutinoside, with concentrations of 632 µg/mL and 136 µg/mL in PAEE and water extract (PAWE), respectively. Other derivatives of quercetin and kaempferol were also observed in the extracts, particularly quercetin glucoside and kaempferol rutinoside. Their concentrations in PAEE extracts were almost five times higher compared to the water extract. In addition, different p-Coumaryl quinic acid isomers were found in significant amounts; however, their total concentration in both extracts did not differ significantly (106 µg/mL in PAEE vs. 98 µg/mL in PAWE).

The extracts from cherry leaves were also a rich source of chlorogenic acids, with a total concentration of 154 µg/mL in the water–ethanol extract (PCEE) compared to 30 µg/mL in the water extract (PCWE). They also contained high concentrations of p-coumaric acid derivatives, including glucosides and isomers of p-coumarylquinic acids (total: 167 µg/mL in PCEE and 98 µg/mL in PCWE). Furthermore, the water–ethanol extract contained significant concentrations of dicaffeoylquinic acids (total: 93 µg/mL). Flavonoids were primarily represented by kaempferol 3-O-rutinoside, followed by quercetin 3-O-rutinoside. The concentration of both flavonoids were five times higher in PCEE.

In turn, phloretin and its derivatives, including glucoside (phloridzin), coumaroyl-5-O-glucoside (belonging to the dihydrochalcone class), and (epi)catechin hexoside, were the predominant constituents of the apple water–ethanol extract (MDEE). Their concentrations were 87, 2584, 87, and 1286 µg/mL, respectively. Interestingly, in the water extract (MDWE), they were found only in low concentrations. Eriodictyol and its hexosides were the most abundant flavonoid compounds. They were present in both extracts; however, similarly to other leaf extracts, water–ethanol was more efficient in isolating these components. The concentrations of aglycone and total hexosides in MDEE were 121 and 131 µg/mL, respectively.

### 2.2. Assessment of Antioxidant Activity

#### 2.2.1. ABTS, DPPH, and FRAP Radical Scavenging

**Figure 1:**
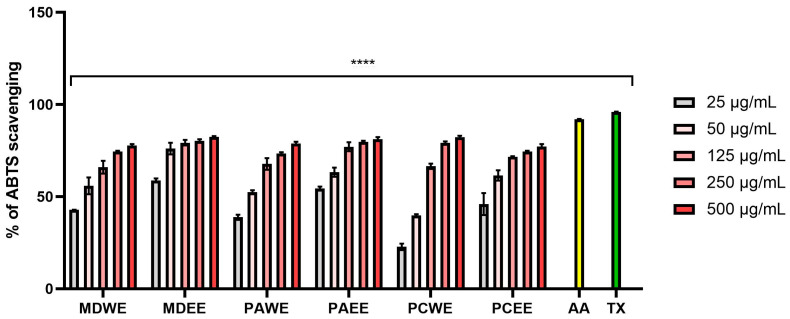
The ability of *M. domestica*, *P. armeniaca*, and *P. cerasus* leaf extracts to scavenge ABTS free radicals at concentrations of 25, 50, 125, 250, and 500 µg/mL. This study used extracts from *M. domestica* leaves (water (MDWE), water–ethanol (MDEE)), extracts from *P. armeniaca* leaves (water (PAWE), water–ethanol (PAEE)), and extracts from *P. cerasus* leaves (water (PCWE), water–ethanol (PCEE)). Ascorbic acid (AA, 100 µg/mL) and Trolox (TX, 100 µg/mL) were used as reference compounds. Data are presented as mean ± SD from three independent experiments, with each sample tested in triplicate **** *p* < 0.0001.

**Figure 2:**
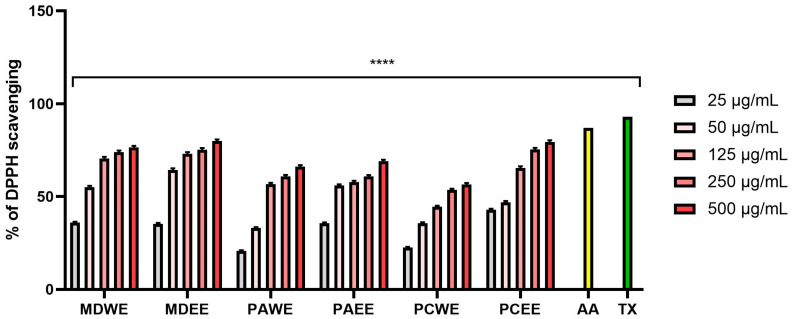
The ability of *M. domestica*, *P. armeniaca*, and *P. cerasus* leaf extracts to scavenge DPPH free radicals at concentrations of 25, 50, 125, 250, and 500 µg/mL. This study used extracts from *M. domestica* leaves (water (MDWE), water–ethanol (MDEE)), extracts from *P. armeniaca* leaves (water (PAWE), water–ethanol (PAEE)), and extracts from *P. cerasus* leaves (water (PCWE), water–ethanol (PCEE)). Ascorbic acid (AA, 100 µg/mL) and Trolox (TX, 100 µg/mL) were used as reference compounds. Data are presented as mean ± SD from three independent experiments, with each sample tested in triplicate **** *p* < 0.0001.

**Figure 3:**
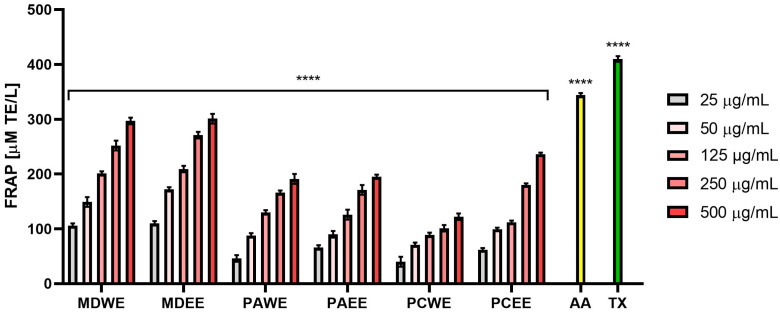
Antioxidant capacity in FRAP assay of *M. domestica*, *P. armeniaca*, and *P. cerasus* leaf extracts at concentrations of 25, 50, 125, 250, and 500 µg/mL. This study used extracts from *M. domestica* leaves (water (MDWE), water–ethanol (MDEE)), extracts from *P. armeniaca* leaves (water (PAWE), water–ethanol (PAEE)), and extracts from *P. cerasus* leaves (water (PCWE), water–ethanol (PCEE)). Ascorbic acid (AA, 100 µg/mL) and Trolox (TX, 100 µg/mL) were used as reference compounds. Data are presented as mean ± SD from three independent experiments, with each sample tested in triplicate **** *p* < 0.0001.

Plant extracts with antioxidant activity play a key role in protecting the skin from oxidative stress, which contributes to the aging process and the development of numerous dermatoses. Free radicals generated as a result of exposure to UV radiation, environmental pollutants, and endogenous factors lead to the degradation of lipid, protein, and DNA structures of skin cells, which results in loss of elasticity, accelerated aging, and increased susceptibility to inflammation and skin diseases [17]. The assessment of antioxidant activity is an important element of research on natural raw materials with potential use in dermatology and cosmetology. Therefore, in this work, the ability to scavenge free radicals (DPPH and ABTS) by the analyzed aqueous and ethanol extracts from the leaves of *Malus domestica*, *Prunus armeniaca*, and *Prunus cerasus* was assessed. The obtained results indicated a stronger antioxidant potential of ethanol extracts compared to aqueous extracts (Figure 1 and Figure 2). The lowest IC50 values in both the ABTS and DPPH tests were observed for apple, apricot, and cherry leaf extracts, respectively. For water extracts, IC50 values ranged from 37.5 µg/mL (for MDWE) to 78.0 µg/mL (for PCWE) in the case of ABTS, and in the case of the test using the DPPH radical, these values ranged from 38.5 µg/mL (for MDWE) to 205.0 µg/mL (for PCWE). In the case of ethanol extracts, the lowest IC50 in the ABTS test was obtained for MDEE (14.5 µg/mL), while the highest was for PCEE (33.5 µg/mL). In the case of the DPPH test, the strongest antioxidant effect was demonstrated by MDEE, for which the IC50 was 32.5 µg/mL, while the weakest was demonstrated by PCEE, reaching an IC_50_ value of 72.5 µg/mL (Table S4). Analyses evaluating the antioxidant properties of the tested extracts by assessing their ability to reduce Fe^3+^ ions indicated an antioxidant effect dependent on the dose, type of extract, and tested plant. The highest concentration of the Fe^2+^–tripyridyltriazine complex was observed for MDWE and MDEE extracts, and the lowest was for PCWE, which indicates the strongest antioxidant properties of apple leaf extracts (Figure 3). The ability to reduce iron ions by transferring electrons and to neutralize DPPH and ABTS radicals observed for all examined extracts indicates that the tested plants can significantly protect cells and biological molecules against the negative effects of oxidative stress.

#### 2.2.2. Evaluation of the Effect on Superoxide Dismutase (SOD) Activity

Plant bioactive compounds such as polyphenols, flavonoids, and phenolic acids can directly scavenge reactive oxygen species, but they can also exert indirect antioxidant effects by modulating endogenous defense systems [18]. One of the key components of the cellular antioxidant machinery is superoxide dismutase, an enzyme that catalyzes the dismutation of superoxide anions to oxygen and hydrogen peroxide [19]. Numerous studies have shown that plant extracts can affect SOD activity, either by upregulating its expression or by stabilizing its enzymatic function under conditions of oxidative stress [20,21]. Since understanding this relationship is essential to elucidating the mechanisms by which plant-derived antioxidants contribute to redox homeostasis and cellular protection, in this work, analyses were performed to determine the effect of the tested samples on SOD activity.

**Figure 4:**
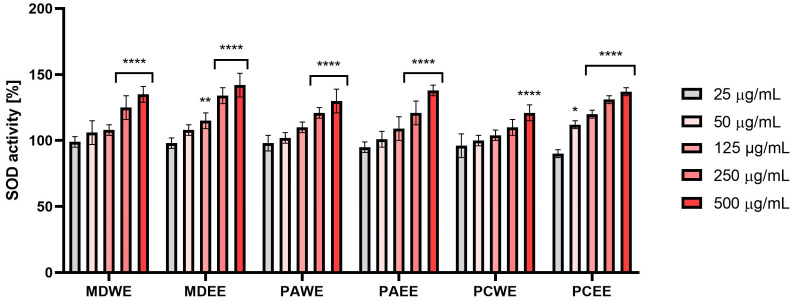
Effect of *M. domestica*, *P. armeniaca*, and *P. cerasus* leaf extracts at concentrations of 25, 50, 125, 250, and 500 µg/mL on superoxide dismutase (SOD) activity. This study used extracts from *M. domestica* leaves (water (MDWE), water–ethanol (MDEE)), extracts from *P. armeniaca* leaves (water (PAWE), water–ethanol (PAEE)), and extracts from *P. cerasus* leaves (water (PCWE), water–ethanol (PCEE)). Data are presented as mean ± SD of three independent experiments, with each sample tested in duplicate. **** *p* < 0.0001, ** *p* = 0.0038, * *p* = 0.0313.

The conducted analyses indicated that all tested extracts were capable of increasing SOD activity, but a statistically significant increase for most extracts was observed only at the two highest tested concentrations (250 and 500 µg/mL). A significant increase in the activity of this enzyme at the concentration of 125 µg/mL was observed only for the ethanolic extracts from apple and cherry leaves. The obtained results therefore suggest that the effect of the extracts from the studied plants on the activity of this enzyme depends on the plant material, the extraction solvent used, and the extract concentration (Figure 4).

So far, there are no literature reports indicating the possibility of inhibition or stimulation of SOD activity by leaf extracts from the three plant species investigated in this study. Considering that this enzyme is involved in maintaining a dynamic balance between the production and elimination of various biologically active molecules with oxidative potential, the ability to influence its activity may be helpful in preventing the toxic effects of free radicals [19]. The modulation of this enzyme’s activity may be related to the action of phytochemicals identified in the studied plants, such as, among others, quinic acid and its derivatives, caffeic acid, quercetin and its derivatives, as well as ferulic acid, which are all compounds with a proven capacity to affect SOD activity [22,23,24,25]. The ability to neutralize DPPH and ABTS radicals, reduce iron ions, and change SOD activity, demonstrated in this study, indicate that the mechanism of antioxidant action of the tested extracts is based on both non-enzymatic and enzymatic antioxidant defense systems. Thanks to the synergistic and interactive action of both of these mechanisms, the ability to neutralize free radicals by the tested plants may be more efficient.

#### 2.2.3. Intracellular ROS Levels in Skin Cells

Oxidative stress, resulting from the excessive production of reactive oxygen species (ROS), is a significant factor leading to skin cell damage. Excess free radicals can cause the lipid peroxidation of cell membranes, damage to structural proteins (e.g., collagen and elastin), and the degradation of nucleic acids, which leads to disorders in the functioning of keratinocytes, fibroblasts, and melanocytes. The use of plant materials rich in natural antioxidants, such as polyphenols, flavonoids, and vitamins C and E, improves skin integrity, increases its resistance to environmental factors and slows down degenerative processes [26]. Consequently, the effective protection of skin cells against oxidative stress contributes to maintaining their functionality, reduces the risk of DNA damage and supports repair mechanisms, which is crucial in the prevention of skin aging and dermatological diseases.

**Figure 5:**
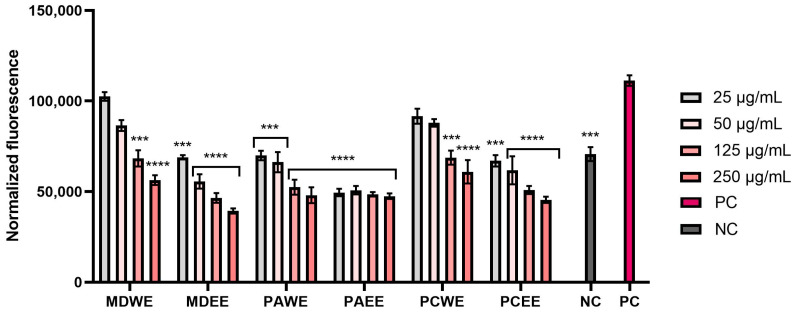
The effect of *M. domestica*, *P. armeniaca*, and *P. cerasus* leaf extracts at the concentrations of 25, 50, 125, and 250 µg/mL on the intracellular level of reactive oxygen species in fibroblasts (HDF cells). The analyses were performed for *M. domestica* leaf extracts (water (MDWE), water–ethanol (MDEE)), *P. armeniaca* leaf extracts (water (PAWE), water–ethanol (PAEE)), and *P. cerasus* leaf extracts (water (PCWE), water–ethanol (PCEE)). Data are presented as mean ± SD from three independent experiments, with each sample tested in triplicate. **** *p* < 0.0001, *** *p* < 0.001.

**Figure 6:**
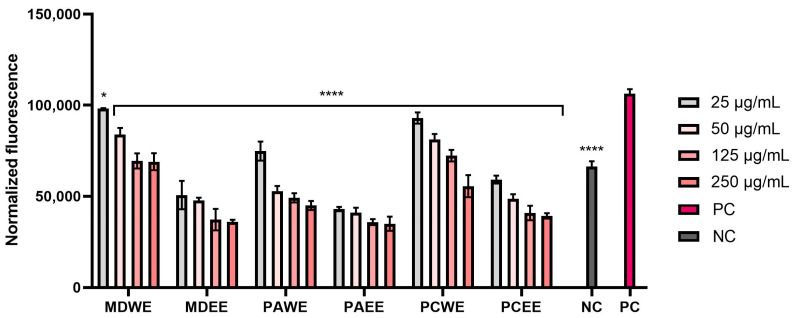
The effect of *M. domestica*, *P. armeniaca*, and *P. cerasus* leaf extracts at the concentrations of 25, 50, 125, and 250 µg/mL on the intracellular level of reactive oxygen species in keratinocytes (HaCaT cells). The analyses were performed for *M. domestica* leaf extracts (water (MDWE), water–ethanol (MDEE)), *P. armeniaca* leaf extracts (water (PAWE), water–ethanol (PAEE)), and *P. cerasus* leaf extracts (water (PCWE), water–ethanol (PCEE)). Data are presented as mean ± SD from three independent experiments, with each sample tested in triplicate. **** *p* < 0.0001, * *p* = 0.0182.

Therefore, the next stage of the study was to assess the effect of the analyzed extracts on the level of reactive oxygen species in skin cells (fibroblasts and keratinocytes) exposed to a factor causing oxidative stress in cells (hydrogen peroxide). The obtained results indicated the ability to reduce the intracellular ROS level, which depended on the extract concentration used, the type of extract, the plant species, and the type of cells (Figure 5 and Figure 6). The analyses showed that ethanol extracts were more effective in lowering intracellular ROS levels compared to aqueous extracts, with a concentration-dependent reduction observed in both HDF and HaCaT cells. The greatest ability to reduce the ROS level in both cell types was observed for MDEE and PAEE. Importantly, in the case of all ethanol extracts, it was observed that the tested samples were able to eliminate the pro-oxidant effect of hydrogen peroxide and reduce the ROS level to a value lower than the negative control (cells not exposed to H_2_O_2_). These results demonstrate the great potential of the analyzed extracts in protecting skin cells against the negative effects of oxidative stress.

The phytochemicals that may be responsible for the ability of the extracts studied are flavonoids, such as quercetin, kaempferol, and catechin, which exhibit strong antioxidant properties due to their ability to donate hydrogen atoms or electrons to free radicals, stabilizing them and terminating the chain reactions of radicals [27,28]. In addition, flavonoids can chelate transition metal ions, preventing the formation of radicals catalyzed by metals, for example, through the Fenton reaction [29]. Another important group of compounds are phenolic acids, including chlorogenic and caffeic acids, which contribute to antioxidant defense by neutralizing free radicals via hydrogen atom transfer (HAT) and single electron transfer (SET) mechanisms [30,31]. The antioxidant potential of phenolic acids is influenced by the number and position of hydroxyl groups in the aromatic ring [32].

Plant-derived antioxidants play a key role in protecting skin cells (keratinocytes, fibroblasts, melanocytes) from oxidative stress induced by various pro-oxidant agents. These compounds reduce intracellular ROS levels, thus preventing lipid peroxidation, DNA damage, and protein oxidation in skin cells [33,34]. The antioxidant effect of the extracts studied may also be due to the fact that flavonoids and phenolic acids activate endogenous antioxidant defense systems by regulating the expression of enzymes such as superoxide dismutase (SOD), catalase (CAT), and glutathione peroxidase (GPx) [35,36]. In addition, they modulate signaling pathways, including the Nrf2-ARE pathway, which enhances the transcription of cytoprotective genes involved in cellular responses to antioxidants [37,38].

### 2.3. Cytotoxicity Assessment

In recent years, there has been an increasing interest in natural plant substances as potential factors supporting skin regeneration and improving its functioning [39]. Leaf extracts from plants such as apple, apricot, or cherry trees show a wide spectrum of biological activity, thanks to which they can affect skin cells. The aim of this study was to assess the effect of apple, apricot, and cherry leaf extracts on skin cell proliferation in vitro. Analyses were carried out for both water and water–ethanol extracts, which were tested at concentrations of 25, 50, 125, and 250 µg/mL. The studies assessed the effect of the tested extracts on epidermal and dermal cells, and the analyses were carried out on keratinocytes (HaCaT) and fibroblasts (HDF). The cytotoxicity of the extracts obtained was assessed using Alamar Blue (AB) and Neutral Red (NR) assays. These tests are popular methods for assessing cell viability after exposure to various types of samples, including plant extracts. The AB test uses resazurin, which changes color due to the metabolic activity of cells, allowing for the assessment of their viability. The NR test, on the other hand, is based on the ability of cells to capture and retain red dye by living cells, which accumulate it in lysosomes, and the change in color intensity after dye extraction is proportional to the number of living cells [40,41].

**Figure 7:**
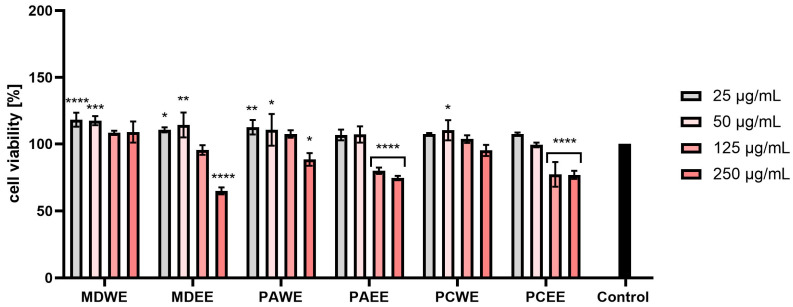
The reduction in resazurin after 24 h exposure to *M. domestica*, *P. armeniaca*, and *P. cerasus* water and water–ethanol extracts in cultured fibroblasts. The analyses were performed for *M. domestica* leaf extracts (water (MDWE), water–ethanol (MDEE)), *P. armeniaca* leaf extracts (water (PAWE), water–ethanol (PAEE)), and *P. cerasus* leaf extracts (water (PCWE), water–ethanol (PCEE)). The control consisted of cells not treated with the extracts. Data are the means ± SDs of three independent experiments, in which each sample was tested in three replicates. **** *p* < 0.0001, *** *p* = 0.0001, ** *p* < 0.01, * *p* < 0.05.

In the AB test conducted on human fibroblasts (HDF), an increase in the viability of these cells was observed after the use of lower concentrations of the tested extracts. The MDWE extract gave higher results than the control in all tested concentrations. In the case of PAWE and PCWE, the viability dropped below the control cells only for the highest concentrations used (250 µg/mL). However, the use of concentrations in the range of 25–125 µg/mL resulted in an increase in the viability of fibroblasts. In the case of other extracts, such as MDEE, PAEE, and PCEE, the two lowest concentrations stimulated the viability of the analyzed cells, and the highest viability was observed for MDWE. Based on the obtained results, a general conclusion can be drawn that, in all the cases tested, lower concentrations had a more beneficial effect on the viability of the tested cells. Increased cytotoxicity at higher concentrations was observed mainly for ethanol extracts (MDEE, PAEE, and PCEE), in which concentrations above 50 µg/mL showed a statistically significant cytotoxic effect (Figure 7).

**Figure 8:**
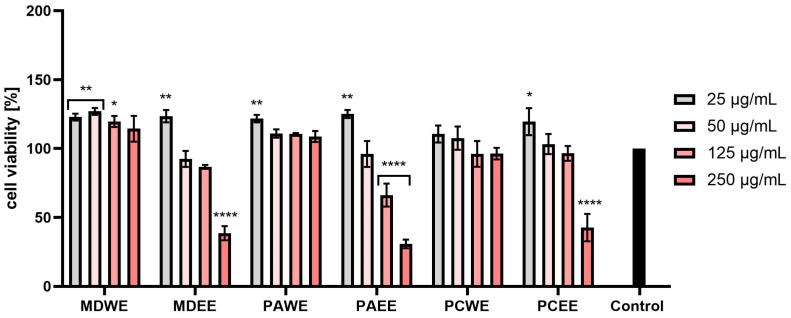
The reduction in resazurin after 24 h exposure to *M. domestica*, *P. armeniaca*, and *P. cerasus* water and water–ethanol extracts in cultured keratinocytes. The analyses were performed for *M. domestica* leaf extracts (water (MDWE), water–ethanol (MDEE)), *P. armeniaca* leaf extracts (water (PAWE), water–ethanol (PAEE)), and *P. cerasus* leaf extracts (water (PCWE), water–ethanol (PCEE)). The control consisted of cells not treated with the extracts. Data are the means ± SDs of three independent experiments, in which each sample was tested in three replicates. **** *p* < 0.0001, ** *p* < 0.01, * *p* < 0.05.

In the case of the AB test performed on HaCaT cells, a similar trend was observed as in the case of HDF cells. MDWE and PAWE extracts stimulated the viability of the tested cells at all applied concentrations. Slightly lower viability values were observed for PCWE, but in the case of this extract, the two highest concentrations resulted in slightly lower viability compared to the control cells. In the case of MDEE, PAEE, and PCEE, the ability to increase cell viability was observed for the lowest tested concentration, while with the increase of the extract concentration in the culture medium, the viability of keratinocytes decreased. For these extracts, in the case of a 250 µg/mL concentration, a decrease in viability of up to about 40% was observed (Figure 8).

**Figure 9:**
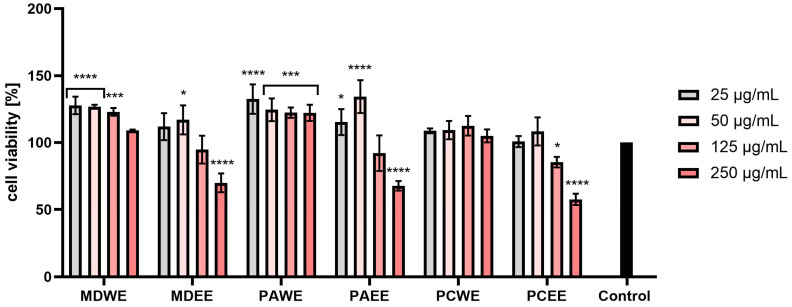
The effect of increasing concentrations after 24 h exposure to *M. domestica*, *P. armeniaca*, and *P. cerasus* water and water–ethanol extracts on Neutral Red dye uptake in cultured fibroblasts. The analyses were performed for *M. domestica* leaf extracts (water (MDWE), water–ethanol (MDEE)), *P. armeniaca* leaf extracts (water (PAWE), water–ethanol (PAEE)), and *P. cerasus* leaf extracts (water (PCWE), water–ethanol (PCEE)).The control consisted of cells not treated with the extracts. Data are the means ± SDs of three independent experiments, in which each sample was tested in three replicates. **** *p* < 0.0001, *** *p* < 0.001, * *p* < 0.05.

The results of the NR tests also indicated a positive effect on the viability of fibroblasts of water extracts (MDWE, PAWE, and PCWE) at all tested concentrations. Apple leaf extracts in the concentration range of 25–125 µg/mL statistically significantly increased the viability of these cells. In the case of MDEE, PAEE, and PCEE, the two lowest concentrations (25 and 50 µg/mL) stimulated viability, while an increase in concentration above 50 µg/mL caused a decrease in the metabolic activity of these cells. Cell viability after the highest concentration decreased most drastically for PCEE and reached a value of about 60% of the viability of control cells. For these extracts, for a concentration of 5.0%, the observed values were as low as about 60% of the control level. Similarly to the AB test, in this case, a stronger cytotoxic effect was also observed in the case of ethanol extracts, especially in the case of the higher of the tested concentrations (Figure 9).

**Figure 10:**
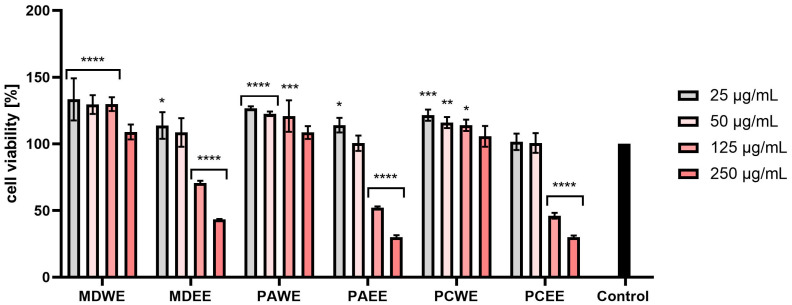
The effect of increasing concentrations after 24 h exposure to *M. domestica*, *P. armeniaca*, and *P. cerasus* water and water–ethanol extracts on Neutral Red dye uptake in cultured keratinocytes. The analyses were performed for *M. domestica* leaf extracts (water (MDWE), water–ethanol (MDEE)), *P. armeniaca* leaf extracts (water (PAWE), water–ethanol (PAEE)), and *P. cerasus* leaf extracts (water (PCWE), water–ethanol (PCEE)). The control consisted of cells not treated with the extracts. Data are the means ± SDs of three independent experiments, in which each sample was tested in three replicates. **** *p* < 0.0001, *** *p* < 0.001, ** *p* = 0.0051, * *p* < 0.05.

The NR test performed on HaCaT cells also indicated a significant increase in the viability of these cells after the use of water extracts from all tested plants. This increase was the highest for the concentration of 25 µg/mL, and in the case of MDWE and PAWE, it exceeded 130%. In the case of ethanol extracts, the two lowest tested concentrations slightly increased cell viability, or this viability remained at the level of control cells. However, after exposure to concentrations above 1.0%, this viability decreased, reaching values below 30% in the case of PAEE and PCEE (Figure 10).

The assessment of biological safety, in particular cytotoxicity towards the human skin cells keratinocytes and fibroblasts, is a key element of research on new substances of plant origin intended for cosmetic and dermatological applications. Keratinocytes, as the most numerous cells of the epidermis, play an important role in creating the skin’s protective barrier and also participate in the immune response and wound healing processes. On the other hand, fibroblasts, located in the dermis, are responsible for the synthesis of extracellular matrix components, such as collagen and elastin, which results in the correct structure, elasticity, and regenerative capacity of the skin [42,43]. As demonstrated in the conducted studies, extracts from apple, apricot, and cherry leaves have a beneficial effect on the proliferation and vitality of keratinocytes and fibroblasts. This effect is particularly important in the context of wound healing processes and counteracting skin ageing [44,45,46]. It seems that the positive effects, especially of aqueous leaf extracts, may be related to the presence of bioactive compounds such as phenolic acids (e.g., gallic acid) and flavonoids. These substances can activate signaling pathways responsible for skin cell proliferation [47,48,49,50], and they also have the ability to stimulate the synthesis of collagen and other structural proteins, which contributes to the improvement of skin condition and regeneration [45,47,51]. Moreover, studies conducted by other researchers have shown that cherry or apricot extracts have a strong protective effect on keratinocytes by reducing cytotoxicity, reducing DNA damage and lowering the level of ROS. This mechanism includes the regulation of protein expression and the activation of signaling pathways, which contribute to the extract’s potential in protecting against oxidative stress [52,53]. It is also worth noting that the presence of ethanol in water–ethanol extracts, although it allows for the effective acquisition of many bioactive compounds, can also lead to undesirable effects, such as cell dehydration or cytotoxic effects. Such properties can limit skin cell proliferation, especially at higher ethanol concentrations [50,51,54]. Despite these limitations, the results of the conducted studies clearly indicate the beneficial effect of *Malus domestica*, *Prunus armeniaca*, and *Prunus cerasus* leaf extracts on the viability of keratinocytes and fibroblasts. The ability to support the proliferation of these cells makes them promising ingredients in the context of use in cosmetic or dermatological products.

### 2.4. Assessment of Antibacterial Activity

The next step of the study was to analyze the antibacterial activity of the tested extracts from fruit tree leaves against selected bacterial strains. The ability of the analyzed samples to inhibit the growth of microorganisms is crucial for their potential use in products intended for direct contact with the skin, because many skin lesions are closely related to the uncontrolled multiplication of microorganisms on the skin surface [55]. The study assessed the effect of water and water–ethanol extracts on the development of bacteria such as *Staphylococcus aureus*, *Staphylococcus epidermidis*, *Bacillus subtilis*, *Staphylococcus capitis*, *Micrococcus luteus*, *Yersinia enterocolitica*, and *Pseudomonas aeruginosa*. The obtained results indicated the antibacterial potential of all the tested extracts. The activity of the tested samples depended on the concentration used, the plant species, and the tested bacterial strain.

The obtained results indicated a slightly stronger activity of water–ethanol extracts compared to water extracts. In the case of analyses of apple leaf extracts, it was shown that these extracts can inhibit the growth of bacteria such as *S. aureus*, *S. epidermidis*, *B. subtilis*, *S. capitis*, and *P. aeruginosa.* Inhibition zones after the use of ethanol extracts reached up to 16 mm [Table 1]. Apricot leaf extracts showed the widest microbiological activity among all three plants tested, as they were able to inhibit the growth of all of the seven bacterial strains tested. This effect was observed in both the water and water–ethanol extracts. The strongest antimicrobial activity of these extracts was observed against bacteria such as *S. aureus* and *S. epidermidis*, for which, in the case of the highest concentration of ethanol extract used (500 µg/mL), growth inhibition zones of 15 and 18 mm were obtained, respectively. The greatest differences in antibacterial activity between water and water–ethanol extracts were observed in the case of cherry leaf extracts, whose antibacterial potential was the weakest of all three plants tested. The water–ethanol extract from the leaves of this plant was unable to inhibit the multiplication of *M. luteus* and *Y. enterocolitica*, while the water extract did not inhibit the multiplication of *S. epidermidis* and *B. subtilis* either. Biologically active compounds present in cherry leaf extracts mainly inhibited the multiplication of *S. aureus*, *S. capitis*, and *P. aeruginosa*, reaching inhibition zones of up to 13 mm.

The MIC values determined for the leaf extracts tested ranged from 150 to 600 µg/mL (Table 2). These values for all plants were lower in the case of extracts obtained using a mixture of water and ethanol (30:70 (*v*/*v*)). In the case of most bacteria, the lowest MIC values were obtained for apple leaf extracts, where the MIC values ranged from 150 to 350 µg/mL. In the case of these extracts, the MIC was not determined for *M. luteus* and *Y. enterocolitica*, because no inhibition of growth of these pathogens was observed in the tested range of extract concentrations (25 to 2000 µg/mL). In the case of apricot leaf extracts, the MIC values ranged from 250 to 600 µg/mL, reaching the highest value in the case of *M. luteus* bacteria. The MIC values determined for cherry leaf extracts ranged from 300 to 450 µg/mL. However, in the case of this plant, the MIC could not be determined (in the range of tested concentrations) for the largest number of tested bacteria, which indicates the narrowest range of antimicrobial activity of the fruits of this plant.

The use of plant extracts containing various types of phytochemicals, including secondary metabolites with proven antibacterial potential, is becoming increasingly popular due to growing concerns about the use of synthetic substances in both cosmetic and pharmaceutical preparations [56]. In addition, their synergistic interaction with various commercially available antibiotics may contribute to enhancing the potential and spectrum of antibiotic action [57,58]. Available scientific articles on the antibacterial activity of *M. domestica* primarily include an assessment of the activity of extracts from the fruit of this plant. Irshad et al. [59] indicated that various types of apple fruit extracts can inhibit the multiplication of bacteria such as *Staphylococcus aureus* and *Salmonella typhi*. These authors also indicated a stronger activity of methanol and ethanol extracts compared to the aqueous extract. Jelodarial et al. indicated antibacterial activity against *Pseudomonas aeruginosa*, *Escherichia coli*, *Klebsiella pneumonia*, and *Staphylococcus epidermidis*, which was dependent on the apple variety tested [7]. Pires et al. [60] indicated that hydromethanolic extracts from dried apple fruits showed the ability to inhibit the growth of *Staphylococcus aureus* and other Gram-positive bacteria, such as *Listeria monocytogenes* and *Enterococcus faecalis*. They also showed the ability to inhibit the growth of Gram-negative bacteria, such as *Escherichia coli* and *Morganella morganii*. Rehab et al. indicated that aqueous extracts can also inhibit the growth of pathogens isolated from animals such as *Staphylococcus aureus*, *Escherichia coli*, *Pseudomonas aerogene*, *Bacillus cereus*, and *Candida albicans* fungi [61]. The antibacterial activity of apple leaves has so far been studied only by Horvacki et al. [62], who compared the possibility of inhibiting the multiplication of such human pathogens as *Escherichia coli*, *Staphylococcus aureus*, and *Bacillus subtilis* by extracts from mesocarp, peel, and leaves of four autochthonous apple varieties. These authors indicated that the highest antibacterial activity against the tested bacterial strains was demonstrated by leaf extracts. Peel extracts showed slightly lower activity, and mesocarp extracts showed only a slight ability to inhibit the growth of *Escherichia coli* and *Bacillus subtillis*. This activity varied depending on the variety used.

*P. armeniaca* is also a plant whose antibacterial properties have been confirmed by other authors, but these studies focused mainly on the properties of extracts from fruits or seeds. Jaya and Lamba indicated the ability of aqueous and ethanol extracts of apricot fruits to inhibit the growth of bacteria such as *Escherichia coli*, *Staphylococcus aureus*, *Proteus vulgaris*, and *Bacillus subtilis*. Additionally, these authors also confirmed the possibility of inhibiting the multiplication of *Candida albicans* by ethanol extracts [63]. Studies conducted by Yigit et al. indicated the antimicrobial activity of methanolic and aqueous extracts of bitter and sweet apricot kernels against clinical isolates of *Escherichia coli*, *Staphylococcus aureus*, *Candida albicans*, and *Candida parapisilosis* [64]. Rashid et al. [65] also indicated the antimicrobial activity of butanol extracts of apricot fruits. They indicated that these extracts can inhibit the growth of such Gram-positive bacteria as *Staphylococcus aureus*, *Staphylococcus epidermidis*, *Streptococcus faecalis*, *Streptococcus pyogenes*, *Micrococcus luteus*, *Bacillus subtilis*, *Corynebacterium diphtheriae*, *Mycobacterium fortuitum*, and *Mycobacterium smegmatis*. These authors also demonstrated the ability to inhibit the growth of Gram-negative bacteria such as *Escherichia coli*, *Salmonella typhi*, *Salmonella paratyphi*, *Shigella dysenteriae*, *Enterobacter aerogenes*, *Klabsiella pneumoniae*, *Pseudomonas aeruginosa*, *Proteus mirabilis*, and *Proteus vulgaris*. The antibacterial properties of leaf extracts from different *P. armeniaca* cultivars were studied only by Sohail et al. [66]. These authors compared the antimicrobial activity of methanolic, aqueous, ethyl acetate, and chloroform extracts and indicated a much stronger activity of the first two types of extracts, which were able to inhibit the multiplication of *Staphylococcus aureus*, *Klebsiella pneumonia*, *Proteus vulgaris*, and *Escherichia coli*.

There are a few reports on the potential antibacterial properties of cherries. In studies on cherry fruit extracts conducted by Coccia et al. [67], the antimicrobial activity of methanolic extracts was assessed. These authors indicated the possibility of inhibiting the growth of such Gram-negative bacteria as *Acinetobacter baumannii*, *Pseudomonas aeruginosa*, *Escherichia coli*, *Proteus mirabilis*, and *Enterobacter cloacae* and Gram-positive bacteria as *Staphylococcus aureus*, *Streptococcus pyogenes*, *Staphylococcus epidermidis*, and *Staphylococcus hominis*. However, the authors of the study indicated that the extracts tested at lower concentrations than the MIC value may stimulate the growth of bacterial biofilm. The antibacterial activity of *P. cerasus* leaves was demonstrated by Berroukche et al., who indicated their ability to inhibit two pathogenic bacterial strains such as Escherichia coli and Staphylococcus aureus [68]. The antibacterial properties of different types of *P. cerasus* leaf and stem extracts were also analyzed by Piccirillo et al. [69]. These authors indicated the antimicrobial activity of these extracts against eight strains of bacteria isolated from clinical, environmental, and food samples. They indicated the possibility of inhibiting the growth of such Gram-positive bacteria as *Bacillus subtilis* and *Staphylococcus aureus*, as well as such Gram-negative bacteria as *Pseudomonas* sp., *Pseudomonas aeruginosa*, *Flavobacterium* sp., *Escherichia coli*, and *Salmonella.*

Studies evaluating the antibacterial activity of the plant extracts studied in this work have expanded the information available so far on the antimicrobial activity of the plants studied. So far, most authors have focused on showing the antibacterial potential mainly of the fruits of the plants studied, and the results presented in this work expand the information on the possibility of inhibiting the multiplication of various pathogenic bacterial strains by extracts obtained from the leaves of the three plants studied. This work has shown the ability to inhibit the growth of bacterial strains that have not been previously used in studies on the antibacterial potential of apple, apricot, and cherry leaves.

The observed differences in the antibacterial activity of the analyzed leaf extracts can be directly linked to the different contents of phytochemicals. The results of the chromatographic identification of biologically active compounds indicate the existence of a relationship between their concentration and effectiveness in limiting the growth of the tested bacterial strains. The compounds that significantly affect the antimicrobial activity of the tested samples are certainly phenolic acids, which are compounds with proven antibacterial properties [70]. The literature data indicate that these acids have activity against both Gram-positive and Gram-negative bacteria. The mechanism of their action may be based on lowering the extracellular pH, changing the potential of the cell membrane and increasing its permeability, which results in disruption of the sodium–potassium pump [71,72]. The antibacterial activity of phenolic acids is strictly dependent on the number and position of substituents in the benzene ring of these compounds. This has an impact on their ability to dissociate. These acids can dissociate at the level of the cell membrane in the case of Gram-positive pathogens, while in the case of Gram-negative bacteria, they act mainly at the cytoplasmic level. Their undissociated, lipophilic form allows them to cross the cell membrane, which causes a decrease in the pH value in the cytoplasm, disruption of the cell membrane structure, and protein denaturation [70,72]. Quinic acid may play an important role. The antibacterial activity of this acid may be related to its influence on the oxidative phosphorylation pathway and changes in the level of glycerophospholipids and fatty acids, which can significantly disrupt the fluidity of bacterial cell membranes. Additionally, this compound may affect ribosome functions and aminoacyl-tRNA synthesis, which leads to protein synthesis disorders. Furthermore, this compound may inhibit the synthesis of l-lysine and peptidoglycan, thus limiting the synthesis of bacterial cell walls and cell division [73]. Phytochemicals that can affect the activity of the tested extracts may also be quercetin and its derivatives. These compounds can inhibit the growth of bacterial cells by changing the permeability of the cell membrane and destroying cell walls. These compounds can also inhibit the synthesis of nucleic acids and affect the expression and activity of various proteins, including enzymes [28,74]. The antibacterial effect may also be related to the presence of catechin derivatives in the extracts, which can act as compounds destroying bacterial walls, cell membranes, and DNA. Moreover, their antibacterial effect may also be related to the suppression of virulence factors and the ability to act synergistically with antibiotics [75,76]. The possibility of inhibiting the multiplication of the tested strains could also be the effect of caffeic acid and its derivatives. These compounds can increase the permeability of cell membranes and stimulate potassium leakage, which can result in damage to bacterial cells [77]. Additionally, studies indicate that the combination of caffeic acid with antibiotics results in synergistic antibacterial activity [78].

In summary, the antibacterial activity of the extracts studied is certainly the result of the action and interactions of various phytochemicals present in the samples obtained. Considering the mechanisms of action of these compounds, the inhibition of the multiplication of the strains studied probably mainly involved the disruption of the membrane potential of bacterial cells, the permeabilization of their inner membrane, the leakage of cellular contents, and the impact on DNA and protein synthesis.

### 2.5. Assessment of Anti-Inflammatory Activity

**Figure 11:**
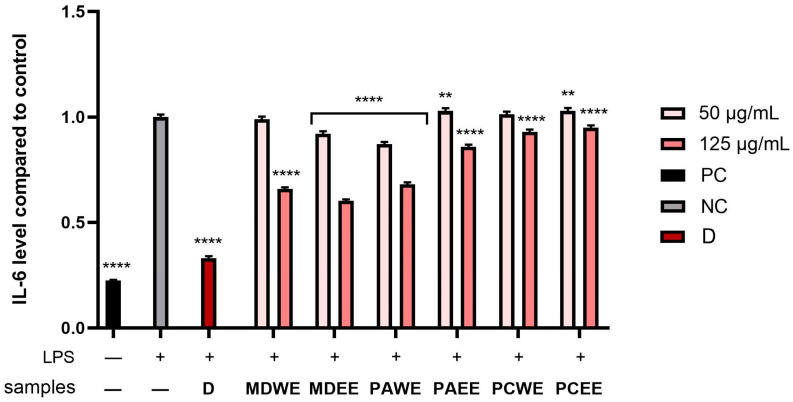
The influence of *M. domestica*, *P. armeniaca*, and *P. cerasus* leaf extracts after exposure to bacterial lipopolysaccharide on interleukin 6 (IL-6) levels. The analyses were performed for *M. domestica* leaf extracts (water (MDWE), water–ethanol (MDEE)), *P. armeniaca* leaf extracts (water (PAWE), water–ethanol (PAEE)), and *P. cerasus* leaf extracts (water (PCWE), water–ethanol (PCEE)) as well as diclofenac (D). Data means ± SD from three independent experiments, in which each sample was tested in duplicate. **** *p* < 0.0001, ** *p* < 0.01.

**Figure 12:**
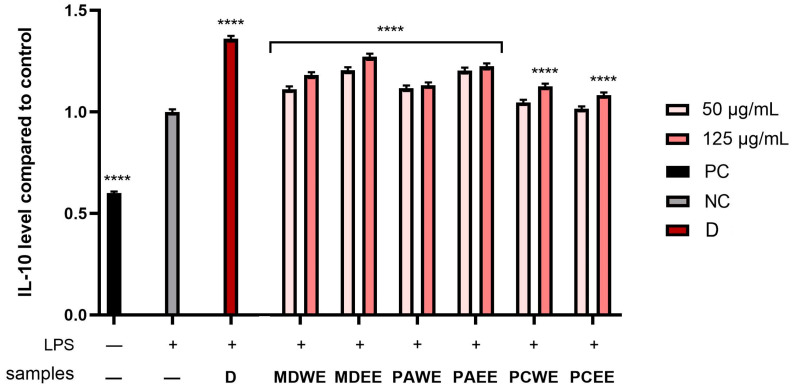
The influence of *M. domestica*, *P. armeniaca*, and *P. cerasus* leaf extracts after exposure to bacterial lipopolysaccharide on interleukin 10 (IL-10) levels. The analyses were performed for *M. domestica* leaf extracts (water (MDWE), water–ethanol (MDEE)), *P. armeniaca* leaf extracts (water (PAWE), water–ethanol (PAEE)), and *P. cerasus* leaf extracts (water (PCWE), water–ethanol (PCEE)), as well as diclofenac (D). Data means ± SD from three independent experiments, in which each sample was tested in duplicate. **** *p* < 0.0001.

**Figure 13:**
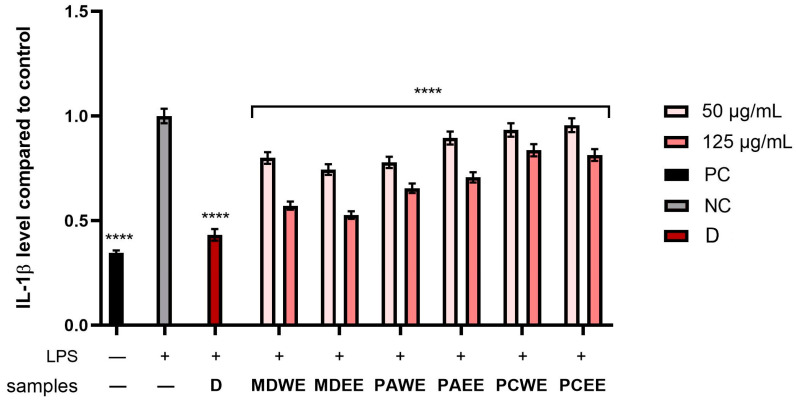
The influence of *M. domestica*, *P. armeniaca*, and *P. cerasus* leaf extracts after exposure to bacterial lipopolysaccharide on interleukin 1β (IL-1β) levels. The analyses were performed for *M. domestica* leaf extracts (water (MDWE), water–ethanol (MDEE)), *P. armeniaca* leaf extracts (water (PAWE), water–ethanol (PAEE)), and *P. cerasus* leaf extracts (water (PCWE), water–ethanol (PCEE)), as well as diclofenac (D). Data means ± SD from three independent experiments, in which each sample was tested in duplicate. **** *p* < 0.0001.

In the next step, the anti-inflammatory potential of *M. domestica*, *P. armeniaca*, and *P. cerasus* leaf extracts was analyzed. For this purpose, the levels of pro-inflammatory interleukins (IL-1β and IL-6) and anti-inflammatory interleukins (IL-10) were assessed in human skin fibroblasts (HDF) after stimulation with bacterial lipopolysaccharide (LPS). The cytokine activity was compared to the control, which included cells not exposed to extracts or LPS. The study also assessed the activity levels of diclofenac (a common anti-inflammatory compound) at a concentation of 10 µg/mL. According to the results shown in Figure 11, Figure 12 and Figure 13, the levels of the enzymes tested increased several times after LPS induction in the control sample, as well as in some plant extract samples. As shown in Figure 11, the level of IL-6 was most strongly reduced after the application of the *M. domestica* extract achieved 0.66 ± 0.008 and 0.60 ± 0.07 fold changes (compared to control without LPS and tested samples) at a 125 µg/mL concentration for ethanolic and aqueous extract (MDWE and MDEE), respectively. Furthermore, PAWE at the same concentration significantly reduced the levels of the cytokine tested, achieving 0.68 ± 0.008 fold changes. In comparison, the anti-inflammatory activity of diclofenac in this study showed 0.33 ± 0.01 fold changes.

This study also determined the level of anti-inflammatory activity of interleukin 10, which is a key mediator in inhibiting the inflammatory response. The results shown in Figure 12 indicate that MDEE at a 125 µg/mL concentration achieved the most favorable effect on the activity of the cytokine, reaching 1.27 ± 0.015 -fold compared to the control, respectively. The significant values in the levels of the tested cytokine were also observed in MDWE at a 125 µg/mL concentration and PAEE at both concentrations tested.

A statistically significant effect of all plant extracts tested on interleukin 1β levels was also observed (Figure 13). Moreover, both aqueous and ethanolic *M. domestica* extracts showed the highest ability to reduce IL-1β, reaching 0.53 ± 0.018 and 0.57 ± 0.02 -fold changes, respectively, at a concentration of 125 µg/mL, which means almost a twofold change in the inhibition of the activity of the tested interleukin compared to the positive sample (LPS-treated cells).

Despite the limited number of publications on the direct effect of the analyzed extracts on the activity of anti-inflammatory cytokines in fibroblasts, there are a number of works indicating their anti-inflammatory properties in other cell lines. One study assessed the profile of phenolic compounds present in apricot leaf extracts and their biological activity, including the ability to modulate the inflammatory response. It was shown that these extracts are a source of hydroxycinnamic acids and flavonols, which are largely responsible for their high antioxidant and anti-inflammatory activity. This study showed significant inhibition of the cyclooxygenase enzymes COX-1 and COX-2, which are key mediators in inflammatory processes [5]. Another study investigated the anti-inflammatory and restorative effects of apple (*M. domestica*) stem cell extract on UVB-induced skin damage in rats. The results showed that the topical application of the extract promoted regeneration of the epidermis and dermis, improving skin barrier function after UVB damage. The extract also reduced the thickness of the epidermis and dermis in the dorsal skin of the animals. In addition, a reduction in polymorphonuclear leukocytes and inflammatory infiltrates was observed after 7 days of treatment [79]. Research on the use of *P. cerasus* extract has shown a reduction in oxidative stress, levels of inflammatory cytokines such as TNF-α and IL-6, and C-reactive protein levels, potentially alleviating conditions such as metabolic disorders and cardiovascular disease. In addition, cherry consumption has been associated with a reduction in inflammation associated with age-related diseases such as arthritis, cardiovascular disease, and cancer [80,81].

The anti-inflammatory effects of *M. domestica*, *P. armeniaca*, and *P. cerasus* leaves can be attributed to their rich content of phenolic compounds, including chlorogenic acid, quercetin, and kaemferol derivatives. These compounds have been shown to inhibit pro-inflammatory mediators and oxidative stress markers, which are key contributors to inflammation. Although direct studies on the anti-inflammatory effects of apricot are limited, the presence of phenolic acids suggests potential benefits. Studies have shown that chlorogenic acid, most abundant in ethanolic extracts of *P. armeniaca* and *P. cerasus* leaves, reduces the production of cytokines such as tumor necrosis factor-α (TNF-α), interleukin-6 (IL-6), and interleukin-1β (IL-1β) [82]. For example, in lipopolysaccharide (LPS)-stimulated macrophages, chlorogenic acid significantly reduced the expression of these cytokines, suggesting its potential to modulate inflammatory responses [83]. On the other hand, kaempferol-3-O-rutonoside, the compound most abundant in PCEE, shows potent anti-inflammatory effects in both in vitro and in vivo models. Anti-inflammatory effects were demonstrated in lipopolysaccharide (LPS)-stimulated RAW 264.7 macrophages and mouse models. It was observed that the inhibition of the production of pro-inflammatory mediators such as nitric oxide (NO), prostaglandin E2 (PGE2), and interleukin-1β (IL-1β) [84]. In addition, it increased the anti-inflammatory cytokine IL-10 and blocked NF-κB phosphorylation, thus demonstrating its anti-inflammatory potential [85]. Furthermore, PAEE contained the highest amount of quercetin-3-O-rutinoside. While direct studies on its effects on fibroblasts are limited, quercitin matabolites have been shown to have anti-inflammatory and antioxidant effects on human keratinocytes (HaCaT cells). They also influence the reduction of pro-inflammatory enzymes such as cyclooxygenase-2 (COX-2) and tumour necrosis factor alpha (TNF-α) in response to UVB radiation or H_2_O_2_ exposure [86]. As shown, the ethanolic extracts of *M. domestica* leaves showed the most favorable properties affecting the activity of IL-6, IL-10, and IL-1β tested. Floridin, a natural dihydrochalcone found mainly in apples was tested for its anti-inflammatory properties in different cell types. The study examined the effects of phloridzin and its aglycone, phloretin, on interleukin-1β (IL-1β)-treated colon myofibroblasts. The results showed that phloretin (10–50 μM) reduced the synthesis of pro-inflammatory molecules such as prostaglandin E_2_ (PGE_2_) and interleukin-8 (IL-8). Although phloridzin was included in the study, its effect was less significant compared to phloretin [87]. Phloretin, which is the aglycone form of phloridzin, has been shown to have anti-inflammatory effects in *P. acnes*-induced skin inflammation. Studies on HaCaT cells have shown that it inhibits PGE2 release and COX-2 expression, suggesting its ability to alleviate skin inflammation [88]. In addition, experiments in mice with 2,4-dinitrochlorobenzene-induced dermatitis showed that phloretin administration (50 and 100 mg/kg, for 21 days) reduces epidermal thickening, mast cell infiltration, and levels of histamine and pro-inflammatory cytokines (IL-6, IL-4, IFN-γ, IL-17A). The mechanism of action is related to the suppression of MAPK and NF-κB pathways in dermal tissues [89].

## 3. Materials and Methods

### 3.1. Chemicals

2,2-Azino-bis-3-ethylbenzothiazoline-6-sulphonic acid (7 mM ABTS solution; Merck KGaA, Darmstadt, Germany), 2,2-diphenyl-1-picrylhydrazyl (DPPH; Merck KGaA, Darmstadt, Germany), 2′,7′-dichlorodihydrofluorescein diacetate (H_2_DCFDA; Merck KGaA, Darmstadt, Germany), acetic acid (CH_3_COOH; ≥99 Warchem, Zakret, Poland), antibiotics (100 U/mL penicillin and 1000 µg/mL streptomycin; Genos, Łódź, Poland), distilled water (H_2_O; Ultrapure Millipore Direct-Q^®^ 3UV-R; Merck, KGaA, Darmstadt, Germany), DMEM (Dulbecco’s Modification of Eagle’s Medium; VWR International, Radnor, PA, USA), ethanol (C_2_H_5_OH; 96%; Warchem, Zakret, Poland), Fetal Bovine Serum (FBS; Biological Industries, Genos, Lodz, Poland), formic acid (MS-grade acetonitrile; Sigma-Aldrich, St. Louis, MO, USA), HRP conjugate (horseradish peroxidase; Elabscience, Houston, TX, USA), hydrogen peroxide (H_2_O_2_; Warchem, Zakret, Poland), LB Agar (Argenta, Poznań, Poland), methanol (CH_3_OH; Warchem, Zakret, Poland), MRS Agar (Argenta, Poznań, Poland), Neutral Red solution (NR; 0.33%; Sigma-Aldrich, Poznan, Poland), phosphate-buffered saline (PBS; pH 7.00 ± 0.05; Genos, Łódź, Poland), potassium persulfate (2.4 mM; Warchem, Zakret, Poland), resazurin sodium salt (RES; Sigma-Aldrich, Poznan, Poland), Substrate Reagent (3,3′,5,5′-tetrametylobenzydyna; Elabscience, TX, USA), Stop Solution (sulfuric acid solution; Elabscience, TX, USA), RIPA (4-nonylphenol; ethoxylated) buffer (EURx; Gdansk, Poland), trypsin-EDTA solution (Sigma-Aldrich, Poznan, Poland), and Trypton Soy Agar (Argenta, Poznań, Poland) were used.

### 3.2. Plant Materials and Extraction Procedure

The plant material was obtained from Naturalny Sklep. The research used apple leaves of the Gala variety, apricot leaves of the Early Orange variety, and cherry leaves of the Nefris variety. Apple leaves were marked by the supplier with voucher number 20240915, apricot leaves with number 20240325, and cherry leaves with 20240702. Two types of extracts were prepared, which were water extract and water–ethanol extract, in a 30:70 ratio. The analyses used extraction with the use of a magnetic stirrer and ultrasound-assistance according to Nizioł-Łukaszewska et al. [90]. To prepare 100 mL of water extract, 5 g of *M. domestica*, *P. armeniaca*, and *P. cerasus* leaves, and 100 mL of distilled water were used. To prepare 100 mL of water–ethanol extract, 5 g of *M. domestica*, *P. armeniaca*, and *P. cerasus* leaves, 30 mL of distilled water, and 70 mL of ethanol were used. Extraction was carried out on a magnetic stirrer (Chemland, Stargard, Poland) for 24 h, and after that time period, the extracts were subjected to ultrasound for 0.5 h. Then, the extracts were filtered using a vacuum pump (Aga Labor, Warsaw, Poland) and stored at 4 °C for further analysis. The yield of extraction for MDWE was 5.06%, for MDEE 6.78%, for PAWE 5.24%, for PAEE 6.89%, for PCWE 5.44%, and for PCEE 6.92%. The obtained extracts from *M. domestica*, *P. armeniaca*, and *P. cerasus* leaves were evaporated at 25 °C in a concentrator (Eppendorf Concentrator Plus, Merck KGaA, Darmstadt, Germany) to obtain stock solutions at a concentration of 5 mg/mL and then were diluted for individual analyses in an appropriate solvent (water or culture medium).

### 3.3. Determination of Biologically Active Compounds

The standards and eluents were purchased from Sigma-Aldrich (St. Louis, MO, USA).

The analytes were separated using an ultra-high-performance liquid chromatography (UHPLC) system from the Infinity II Series, equipped with a DAD detector and an Agilent 6224 ESI/TOF mass detector (Agilent Technologies, Santa Clara, CA, USA). The chromatographic system utilized a Kinetex C18 reversed-phase column 100Å (Phenomenex, Torrance, CA, USA) with dimensions of 150 mm × 2.1 mm and a particle size of 1.7 µm. The chromatographic conditions were described previously [91].

Chromatographic analysis was performed within the wavelength range from 200 to 600 nm. The MS parameters were as follows: drying gas temperature of 325 °C, flow rate of 8 L/min, nebulizer pressure of 30 psi, capillary voltage of 3500 V, fragmentor voltage of 220 V, and skimmer voltage of 65 V [92].

Identification was carried out by comparing the obtained results with available reference standards. In cases where standards were unavailable, identification was based on the literature data. Quantification was performed using calibration curves generated from standard solutions of the identified compounds.

### 3.4. Determination of Antioxidant Properties

#### 3.4.1. ABTS Scavenging Assay

The antioxidant properties of the tested extracts from the leaves of *M. domestica*, *P. armeniaca*, and *P. cerasus* were assessed using the ABTS cationic radical test [93]. Briefly, a mixture of this radical at a concentration of 7 mM (Merck, Darmstadt, Germany) and 2.4 mM potassium persulfate solution (Warchem, Zakręt, Poland) was prepared first. Both solutions were then mixed in a 1:1 ratio. After 24 h of incubation of this mixture at a temperature of about 22–24 °C, the solution was diluted in PBS buffer to obtain an absorbance value of 1.0 ± 0.07 at λ = 734 nm. Then, individual concentrations of the obtained extracts (in the concentration range from 25 to 500 µg/mL) were mixed with 7 mM of ABTS solution, and the absorbance of the samples was measured at λ = 734 using a UV/VIS spectrophotometer (Thermo Fisher Scientific, Waltham, MA, USA). The positive controls in this method were Trolox (Merck KGaA, Darmstadt, Germany) and ascorbic acid (Warchem, Zakręt, Poland). The negative control was distilled water. Individual samples of the tested extracts without ABTS were blanks. The results are presented as the percentage of ABTS scavenging compared to the control (Equation (1)). Three independent experiments were conducted to assess the level of ABTS radical scavenging, in which each concentration of the tested extracts was measured three times.

(1) $$\%\;ABTS\;scavenging=\left(1-\left(\frac{Abs\;sample}{Abs\;control}\right)\right)\times 100$$

#### 3.4.2. DPPH (1,1-Diphenyl-2-Picrylhydrazyl) Radical Scavenging Assay

The antioxidant activity of the tested leaf extracts was also assessed using the DPPH (1,1-diphenyl-2-picrylhydrazyl) radical [94]. The ability to scavenge this radical was tested by placing appropriate concentrations of the tested samples (25–500 µg/mL) into individual wells on a 96-well plate. Then, 100 µL of a methanolic DPPH solution (0.4 mM, Merck KGaA, Darmstadt, Germany) was added to each well. The prepared plates were mixed on an orbital shaker for 15 min. The positive controls were Trolox and ascorbic acid, and the negative control was distilled water. The tested concentrations of extracts without DPPH were blanks. After shaking, the plate was placed in a plate reader, and absorbance was measured at λ = 517 nm using a UV/VIS spectrophotometer (Thermo Fisher Scientific, Waltham, MA, USA). The results were presented as percentage of DPPH radical scavenging (Equation (2)). Three independent experiments were performed in the study, in which each concentration of extracts was analyzed in triplicate.

(2) $$\%\;DPPH\;scavenging=\frac{Abs\;control-Abs\;sample}{Abs\;control}\times 100$$

#### 3.4.3. Determination of Ferric Reducing Antioxidant Power (FRAP Assay)

The antioxidant capacity of the tested extracts was also determined using a spectrophotometric FRAP assay. Analyses were performed based on the methodology described by Benzie and Strain [95]. First, a FRAP mixture consisting of 0.3 M acetate buffer, 0.01 M tripyridyltriazine (TPTZ, Merck KGaA, Darmstadt, Germany), and 0.02M FeCl_3_ × 6H_2_O mixed in a 10:1:1 ratio was prepared. Then, 180 μL of the FRAP mixture and 20 μL of individual dilutions (25–500 µg/mL) of the tested leaf extracts were added to the wells of a 96-well plate. The blank sample was a mixture of 180 μL of the FRAP mixture and 20 μL of distilled water. The positive controls were Trolox and ascorbic acid at a concentration of 100 µg/mL mixed with 180 μL of the FRAP mixture. The prepared plates were incubated for 20 min, after which absorbance measurements were taken at λ = 593 nm using a plate reader (BioTek Synergy SH1MG, Agilent Technologies, Santa Clara, CA, USA). The Trolox (Merck KGaA, Darmstadt, Germany) standard curve was prepared in a range of concentrations (0–1000 μM). Three independent experiments were performed in the study, in which each concentration of the tested extracts and positive samples was analyzed three times. The results were calculated based on the calibration curve formula and are expressed in Trolox equivalents (μM TE/L).

#### 3.4.4. Determination of Superoxide Dismutase (SOD) Activity

To assess the potential mechanism of antioxidant activity of the tested samples, their effect on superoxide dismutase activity was assessed according to the protocol provided by the manufacturer (ab65354, Abcam, Cambridge, UK). Analyses were performed for leaf extract samples in the concentration range of 25–500 µg/mL. Recombinant human SOD 1 protein (ab112193, Abcam, Cambridge, UK) was used to prepare the standard curve. SOD activity was assessed in 96-well flat-bottomed, transparent plates. First, 200 μL of 2-(4-iodophenyl)-3-(4-nitrophenyl)-5-(2,4-disulfo-phenyl)-2H-tetrazolium monosodium salt (WST) working solution was added to each well. Then, 20 μL of enzyme working solution (EWS) and 20 μL of analyzed samples at individual concentrations were added. Three different blank samples were also prepared according to the kit manufacturer’s recommendations. Blank sample 1 was prepared by adding 20 μL of EWS and 20 μL of ddH_2_O to the wells, blank sample 2 was created by adding 20 μL of dilution buffer (DB) and 20 μL of the analyzed samples, and blank sample 3 was created by adding 20 μL of DB and 20 μL of ddH_2_O. The plate was thoroughly mixed on an orbital shaker and incubated in the dark at 37 °C for 20 min. The absorbance of individual wells was then measured at λ = 450 nm using a microplate reader (FilterMax F5, Thermo Fisher, Waltham, MA, USA). The analyses consisted of three independent experiments, in which each concentration of the tested extracts was tested in duplicate according to the manufacturer’s instructions. The ability to inhibit SOD activity was calculated from the following equation:

(3) $$\%\;SOD\;Activity=\frac{\left(A_{blank1}-A_{blank3}\right)-(A_{sample}-A_{blank2})}{\left(A_{blank1}-A_{blank3}\right)}\times 100$$

#### 3.4.5. Detection of Intracellular Levels of Reactive Oxygen Species (ROS)

The antioxidant activity of the extracts tested was also assessed by measuring the intracellular level of reactive oxygen species (ROS) in human fibroblasts and keratinocytes using the fluorogenic probe H_2_DCFDA [96]. Briefly, both cell lines were seeded separately on black 96-well plates at a density of 1 × 10^4^ and cultured for 24 h in DMEM medium at 37 °C. Then, the medium was aspirated from the wells and replaced with individual concentrations of the six leaf extracts tested (in the concentration range of 25–250 µg/mL) dissolved in DMEM. The plates prepared in this way were incubated in an incubator for 24 h. After this time, the extract solutions were removed, and the cells were treated with 10 µM H_2_DCFDA (Sigma Aldrich, Sant Louis, MO, USA) dissolved in DMEM without the addition of FBS serum. To induce oxidative stress in cells, hydrogen peroxide (H_2_O_2_, Merck KGaA, Darmstadt, Germany) was also added to each well at a final concentration of 500 µM. Negative controls were cells (separately HDF and HaCaT) not treated with H_2_O_2_ or the tested extracts, and positive controls were cells treated only with 500 µM of hydrogen peroxide dissolved in DMEM. ROS levels in in vitro skin cells treated with the tested samples were assessed spectrophotometrically by measuring fluorescence at an excitation wavelength of λ = 485 nm and an emission wavelength of λ = 530 nm after 60 min of incubation with the fluorescent probe. Measurements were performed using a microplate reader (FilterMax F5, Thermo Fisher Scientific, Waltham, MA, USA). The analyses were performed using three independent experiments, in which each concentration of extracts was tested in triplicate.

### 3.5. Cytotoxicity Analysis

#### 3.5.1. Cell Culture

The analysis of cytotoxicity of the tested extracts and their effect on the intracellular level of reactive oxygen species was performed on two skin cell lines, human fibroblasts (HDF) and keratinocytes (HaCaT). The levels of pro- or anti-inflammatory cytokines were assessed on HDF cells. Both lines used were purchased from CLS Cell Lines Service (Eppelheim, Germany). The cells were cultured in DMEM (Dulbecco’s Modified Eagle’s Medium, Biological Industries, Cromwell, CO, USA) additionally supplemented with sodium pyruvate, L-glutamine, glucose (4.5 g/L), and fetal bovine serum at a final concentration of 10.0% (Genos, Łódź, Poland). To prevent contamination of the medium, it was additionally supplemented with 1.0% antibiotics (100 U/mL penicillin and 1000 μg/mL streptomycin, Thermo Fisher Scientific, Waltham, MA, USA). Cells were cultured in culture flasks (Googlab Scientific, Rokocin, Poland) with a growth surface of 75 cm^2^. The culture was carried out in an incubator at 37 °C, 95.0% air, and 5.0% carbon dioxide. Cells were passaged using trypsinization after reaching approximately 70–80.0% confluence.

#### 3.5.2. Alamar Blue (AB) and Neutral Red (NR) Assays

Two tests were used to assess the cytotoxicity of the tested leaf extracts. The first one was based on the measurement of the level of resazurin sodium salt reduction (Alamar Blue (AB) test, Merck KGaA, Darmstadt, Germany), while the second one allowed the assessment of the ability of cells to accumulate Neutral Red dye in lysosomes (Neutral Red (NR) test, Merck KGaA, Darmstadt, Germany). The studies were carried out based on the methodology described earlier by Ziemlewska et al. with minor modifications [97]. Resazurin reduction was assessed on cells grown on 96-well black flat-bottom plates, while Neutral Red incorporation was assessed on transparent flat-bottom plates (Googlab Scientific, Rokocin, Poland). After 24 h from seeding the cells on the plates, the cells were exposed to the tested extracts at concentrations from 25 to 250 µg/mL for 24 h. Control cells were both fibroblasts and keratinocytes (separately) not exposed to the tested samples and cultured in complete DMEM medium. After 24 h, the extract solutions dissolved in DMEM were aspirated and replaced with 60 µM of resazurin solution. Then, the cells were incubated with this dye for 2 h, after which fluorescence was measured in individual wells at λ = 570 nm using a microplate reader (Thermo Fisher Scientific, Waltham, MA, USA). In the case of the test using Neutral Red, after aspirating the individual concentrations of extracts from the wells (analogous to the AB test), 40 µg/mL of Neutral Red solution was added to each well. Then, the cells were incubated with this dye in an incubator for 2 h. In the next step, the cells (both HDF and HaCaT) were washed twice with phosphate-buffered saline (PBS, Genos, Lodz, Poland). Then, to release the dye from the lysosomes, a destaining buffer consisting of ethanol, acetic acid, and water in a ratio of 50%:1%:49% was added to the wells. The prepared plates were shaken on an orbital shaker for 15 min, after which the absorbance of the solution in individual wells was measured at λ = 540 nm using a microplate reader (ThermoFisher Scientific, Waltham, MA, USA). Cell viability was calculated assuming that the viability of control cells (untreated with extracts) was 100%. Three independent experiments were performed in the analyses, testing each extract concentration in triplicate.

### 3.6. Assessment of Antibacterial Activity

#### 3.6.1. Disk Diffusion Assay

The impact of the analyzed leaf extracts on the growth of pathogenic bacteria was assessed using the disc diffusion method described earlier by Nizioł-Łukaszewska et al. [90]. The strains that were analyzed were obtained from the American Type Culture Collection (Manassas, VA, USA). Antibacterial analyses were performed on *Staphylococcus aureus* ATCC BAA-2312, *Staphylococcus capitis* ATCC^®^ 146™, *Staphylococcus epidermidis* ATCC^®^ 49134™, *Bacillus subtilis* ATCC^®^ 19659, *Micrococcus luteus* ATCC^®^ 10240™, *Yersinia enterocolitica* ATCC^®^ 27729, and *Pseudomonas aeruginosa* ATCC^®^ 35032. In the first step, 10 mL of agar medium appropriate for individual bacterial strains was poured onto sterile Petri dishes. The media used in the study were tryptone soy agar, nutrient agar, LB agar, and MRS agar obtained from Argenta (Poznań, Poland). Then, individual bacterial strains were inoculated onto previously prepared media on Petri dishes. The inoculum density was 5 × 10^7^ CFU (colony forming units)/mL. In the next step, sterile filter paper discs were soaked with various concentrations of the tested extracts (50, 250, and 500 µg/mL), which had been previously sterilized through membrane filters (0.22 µm). The soaked discs were placed on the surface of solidified media on Petri dishes, on which individual bacterial strains had been previously inoculated. A sterile disc soaked in sterile deionized water was used as a negative control. Positive controls were appropriate antibiotics inhibiting the growth of the tested microorganisms. Vancomycin was used as the reference antibiotic for *S. aureus*, *S. epidermidis*, *S. capitis*, and *B. subtilis*. For *M. luteus*, erythromycin was applied, while ciprofloxacin served as the reference antibiotic for *Y. enterocolitica* and *P. aeruginosa*. The prepared plates were placed in an incubator and cultured at 35 ± 2 °C for 24 h. Then, the growth inhibition ability of the tested strains was assessed by measuring the diameter of the growth inhibition zone.

#### 3.6.2. Determination of Minimum Inhibitory Concentrations (MIC)

The antibacterial activity of the tested samples was also assessed by determining the minimum inhibitory concentration (MIC). For this purpose, the tested extracts were evaporated and then dissolved in sterile distilled water to obtain a stock with a concentration of 10 mg/mL. The MIC was determined using the microdilution technique in broth with p-iodonitrotetrazolium violet (INT, Merck KGaA, Darmstadt, Germany) as a growth indicator, according to the procedure described by Eloff [98]. The analysis was started by multiple dilutions in broth to obtain concentrations from 25 to 2000 μg/mL. Such prepared dilutions of the tested extracts (in triplicate) were placed in 96-well microplates with a flat bottom. Then, a suspension of individual bacterial strains (5 × 10^4^ colony-forming units (CFU)) was added to each well. For each of the tested microorganisms, a sterility control well, an antibiotic well, and a growth control well were also prepared. The plates were subjected to 24 h incubation at 37 °C. After this time, 40 μL of INT solution at a concentration of 0.4 mg/mL was added to each well and incubated for 30 min. The MIC value was determined as the concentrations of individual leaf extracts that completely inhibited the growth of the tested bacterial strains. As part of the analyses, three independent experiments were performed.

### 3.7. Assessment of Anti-Inflammatory Activity

To evaluate the anti-inflammatory effects of extracts obtained from *M. domestica*, *P. armeniaca*, and *P. cerasus* leaves, the levels of IL-1β, IL-6, and IL-10 were measured in fibroblasts (HDF) treated with bacterial lipopolysaccharide (LPS, 10 µg/mL) derived from Escherichia coli O111:B4 for 24 h. Simultaneously, cells cultured in 6-well plates were treated with test extracts at concentrations of 50 and 125 µg/mL. This study also assessed the activity of levels of diclofenac at a concentation of 10 µg/mL. After 24 h of incubation of cells exposed to LPS and the analyzed samples, DMEM was removed from the wells. The wells were then washed with PSB, and 150 µL of RIPA buffer was added to each well, leading to cell lysis. The samples thus prepared were analyzed using commercial ELISA kits (Elabscience Biotechnology Inc., Houston, TX, USA) according to the manufacturer’s instructions. Absorbance was measured at 450 nm using a microplate reader (FilterMax F5, Thermo Fisher Scientific, Waltham, MA, USA). Cells that were neither treated with LPS nor treated with extracts were negative controls (NC), while cells treated with LPS but not treated with extracts were positive controls (PC).

### 3.8. Statistical Analysis

Statistical analysis was started by checking whether the data obtained during the conducted analyses met the assumption of normality and were subject to a normal distribution. For this purpose, the Shapiro–Wilk test was performed. The values of the analyzed parameters were presented as mean ± standard deviation (SD). Then, statistical significance was assessed using two-way analysis of variance (ANOVA) followed by Dunnett’s post hoc test for group comparisons. Statistical significance was considered as **** *p* < 0.0001, *** *p* < 0.001, ** *p* < 0.01, and * *p* < 0.05 compared to the control group. GraphPad Prism 8.4.3 (GraphPad Software, Inc., San Diego, CA, USA) was used to perform statistical analyses.

## 4. Conclusions

The conducted studies clearly demonstrated that extracts obtained from the leaves of apple, apricot, and cherry trees exhibit significant biological activity, including antioxidant, anti-inflammatory, and antibacterial properties. All the tested extracts showed considerable antioxidant potential, with the ethanol-based extracts displaying the highest activity, indicating their greater capacity to neutralize free radicals. The observed antioxidant effects of the tested extracts are mediated through both non-enzymatic mechanisms, such as free radical scavenging and metal ion reduction, as well as enzymatic pathways involving the modulation of SOD activity. Among the analyzed samples, apple leaf extracts exhibited the strongest anti-inflammatory activity, while apricot leaf extracts showed the broadest antibacterial spectrum, effectively inhibiting the growth of all seven tested pathogenic strains. Additionally, at lower concentrations, these extracts did not exhibit cytotoxicity towards human skin cells in vitro and may provide protection against oxidative stress induced by hydrogen peroxide, suggesting their potential protective and regenerative effects on the skin.

The findings emphasize the importance of utilizing plant-derived waste materials, such as fruit tree leaves, as a valuable source of bioactive compounds for innovative cosmetic formulations. As shown in this work, the use of different extraction systems allows for obtaining different amounts and types of phytochemicals, which significantly affects the profile of biological activity of the extracts. The variety of solvents used allows for the selective isolation of specific classes of active compounds, which creates opportunities to optimize the extraction process for specific functional properties of the final product. The obtained results confirm the validity of further research on the use of waste plant materials as a sustainable source of active ingredients for the cosmetics industry.

The obtained data constitute a promising basis for further work on the use of these extracts in products with cosmetic potential. The results suggest the good biocompatibility of these extracts (in particular, no toxicity to skin cells), which may indicate their potential use in cosmetic or dermatological products, especially in formulations for sensitive or problematic skin. However, it is important to highlight a number of limitations that may affect the interpretation of the results and the subsequent application of the extracts. The phytochemical composition of plant leaves is strongly influenced by environmental conditions, such as crop location, soil type, sunshine, harvesting season, and drying process used. These differences can lead to variability in the content of active compounds, making the standardization and comparison of results difficult. The study used in in vitro models, which, although commonly used as a first step in assessing safety and biological activity, does not fully reflect the complex mechanisms occurring in a living organism. Factors such as skin metabolism, the microbiome, the immune system, or allergic reactions cannot be taken into account in vitro. Therefore, further in vivo studies—both in animal models and in clinical trials—are needed in the next step to confirm the safety and efficacy of the extracts on human skin. In addition, an important aspect is to assess the stability of the extracts in the finished cosmetic formulation, which may have an impact on their actual performance after application. Potential interactions with other formulation ingredients, such as preservatives, emulsifiers, or formulation pH, may also modify the activity of the extracts and require further analysis.

In future studies, it is planned to deepen the analysis of the mechanisms of action of extracts, including the use of molecular techniques such as Western blot, to assess the expression of selected proteins involved in inflammatory processes and oxidative stress. In addition, the studies will be extended to analyses on skin cancer cells and 3D skin models. In the longer term, it will also be advisable to determine the stability and bioavailability of active compounds in physiological conditions and their potential synergistic activity in combination with other natural ingredients.

## Figures and Tables

**Table 1:** Antibacterial activity of the tested *M. domestica*, *P. armeniaca*, and *P. cerasus* leaf extracts expressed as the diameter of the average inhibition zone (mm).

Bacteria	Plant Species	Zone of Inhibition [mm]					
		Water Extract			Water–Ethanol Extract		
		50 µg/mL	250 µg/mL	500 µg/mL	50 µg/mL	250 µg/mL	500 µg/mL
*Staphylococcus aureus*	*M. domestica*	5	7	9	7	10	13
	*P. armeniaca*	4	7	10	6	9	15
	*P. cerasus*	7	12	9	4	7	10
*Staphylococcus epidermidis*	*M. domestica*	4	7	11	6	9	14
	*P. armeniaca*	3	6	8	8	11	18
	*P. cerasus*	nd	nd	nd	nd	5	8
*Bacillus subtilis*	*M. domestica*	5	7	10	5	8	11
	*P. armeniaca*	nd	5	7	6	9	10
	*P. cerasus*	nd	nd	nd	4	10	10
*Staphylococcus capitis*	*M. domestica*	nd	12	9	6	16	12
	*P. armeniaca*	nd	5	7	4	8	10
	*P. cerasus*	4	9	7	6	13	10
*Micrococcus luteus*	*M. domestica*	nd	nd	nd	nd	nd	nd
	*P. armeniaca*	nd	nd	5	nd	8	10
	*P. cerasus*	nd	nd	nd	nd	nd	nd
*Yersinia enterocolitica*	*M. domestica*	nd	nd	nd	nd	nd	nd
	*P. armeniaca*	nd	4	7	6	8	9
	*P. cerasus*	nd	nd	nd	nd	nd	nd
*Pseudomonas aeruginosa*	*M. domestica*	nd	5	7	6	8	11
	*P. armeniaca*	nd	4	7	5	8	10
	*P. cerasus*	nd	5	7	5	8	11

nd—not detected.

**Table 2:** Minimum inhibitory concentrations (MIC) of *M. domestica*, *P. armeniaca*, and *P. cerasus* leaf extracts against the tested bacteria.

Bacteria	Plant Species	Minimum Inhibitory Concentration MIC [µg/mL]	
		Water Extract	Water–Ethanol Extract
*Staphylococcus aureus*	*M. domestica*	350	250
	*P. armeniaca*	350	250
	*P. cerasus*	300	350
*Staphylococcus epidermidis*	*M. domestica*	250	250
	*P. armeniaca*	350	250
	*P. cerasus*	nd	400
*Bacillus subtilis*	*M. domestica*	350	250
	*P. armeniaca*	400	250
	*P. cerasus*	nd	300
*Staphylococcus capitis*	*M. domestica*	300	150
	*P. armeniaca*	400	300
	*P. cerasus*	400	350
*Micrococcus luteus*	*M. domestica*	nd	nd
	*P. armeniaca*	600	450
	*P. cerasus*	nd	nd
*Yersinia enterocolitica*	*M. domestica*	nd	nd
	*P. armeniaca*	400	300
	*P. cerasus*	nd	nd
*Pseudomonas aeruginosa*	*M. domestica*	350	250
	*P. armeniaca*	400	300
	*P. cerasus*	450	300

nd—not detected.

## Data Availability

The data presented in this study are available on request from the corresponding author.

## Supplementary Materials

The following supporting information can be downloaded at: https://www.mdpi.com/article/10.3390/molecules30102085/s1, Figure S1. Base peak chromatogram of leaf extracts from *P. armeniaca* (blue line), *P. cerasus* (red line), and *M. domestica* (green line). Table S1. Quantitative analysis of *P. armeniaca* leaf extract performed using UHPLC/DAD/ESI-MS. The values represent means ± standard deviation (SD) of triplicate. Table S2. Quantitative analysis of *P. cerasus* leaf extract performed using UHPLC/DAD/ESI-MS. The values represent means ± standard deviation (SD) of triplicate. Table S3. Quantitative analysis of *M. domestica* leaf extract performed using UHPLC/DAD/ESI-MS. The values represent means ± standard deviation (SD) of triplicate. Table S4. ABTS and DPPH radical scavenging IC_50_ values for *M. domestica*, *P. armeniaca* and *P. cerasus* leaf extracts. The values were determined for extracts from *M. domestica* leaves (water (MDWE), water-ethanol (MDEE)), extracts from *P. armeniaca* leaves (water (PAWE), water-ethanol (PAEE)) and extracts from *P. cerasus* leaves (water (PCWE), water-ethanol (PCEE)). Values are means ± standard deviation (SD) of triplicates.
