# Supplementary material for: Comparison of Cytotoxicity and Antioxidant, Antibacterial, and Anti-Inflammatory Activity of Aqueous and Ethanolic Extracts from Malus domestica, Prunus armeniaca, and Prunus cerasus Leaves
Source: Molecules. 2025 May 8;30(10):2085. doi: 10.3390/molecules30102085 (PMC12114224; doi:10.3390/molecules30102085)
Supplement: Supplementary file 1 [file molecules-30-02085-s001.zip › molecules-3590705-supplementary.pdf]

molecules

Article

Comparison of cytotoxicity and antioxidant, antibacterial and anti-inflammatory activity of aqueous and ethanolic extracts from *Malus domestica*, *Prunus armeniaca* and *Prunus cerasus* leaves

Martyna Zagórska-Dziok [1], Aleksandra Ziemlewska [1], Magdalena Wójciak [2] , Ireneusz Sowa [2] ,Ewa Wąsik-Szczepanek [3] and Zofia Nizioł-Łukaszewska [1,*]

[1] Department of Technology of Cosmetic and Pharmaceutical Products, Medical College, University of Information Technology and Management in Rzeszow, Sucharskiego 2, 35-225 Rzeszow, Poland; mzagorska@wsiz.edu.pl (M.Z.-D.); aziemlewska@wsiz.edu.pl (A.Z.); zniziol@wsiz.edu.pl (Z.N.-Ł.)
[2] Department of Analytical Chemistry, Medical University of Lublin, Aleje Raclawickie 1, 20-059 Lublin, Poland; magdalena.wojciak@umlub.pl (M.W.); ireneusz.sowa@umlub.pl (I.S.)
3 Chair and Department of Hematooncology and Bone Marrow Transplantation, Medical University of Lublin, Staszica 11, 20-081 Lublin, Poland; ewa.wasik-szczepanek@umlub.pl (E.W.-S)

* Correspondence: zniziol@wsiz.edu.pl (Z.N.-Ł.)

Academic Editors: Artur M. S. Silva, Hinanit Koltai and Jean-Marc Sabatier

Received: 31 March 2025
Revised: 3 May 2025
Accepted: 6 May 2025
Published: 8 May 2025

Citation: Zagórska-Dziok, M.; Ziemlewska, A.; Wójciak, M.; Sowa, I.; Wąsik-Szczepanek, E.; Nizioł-Łukaszewska, Z. Comparison of Cytotoxicity and Antioxidant, Antibacterial and Anti-Inflammatory Activity of Aqueous and Ethanolic Extracts from *Malus domestica*, *Prunus armeniaca* and *Prunus cerasus* Leaves. *Molecules* **2025**, *30*, 2085. https://doi.org/10.3390/molecules30102085

Copyright: © 2025 by the authors. Licensee MDPI, Basel, Switzerland. This article is an open access article distributed under the terms and conditions of the Creative Commons Attribution (CC BY) license (https://creativecommons.org/licenses/by/4.0/).

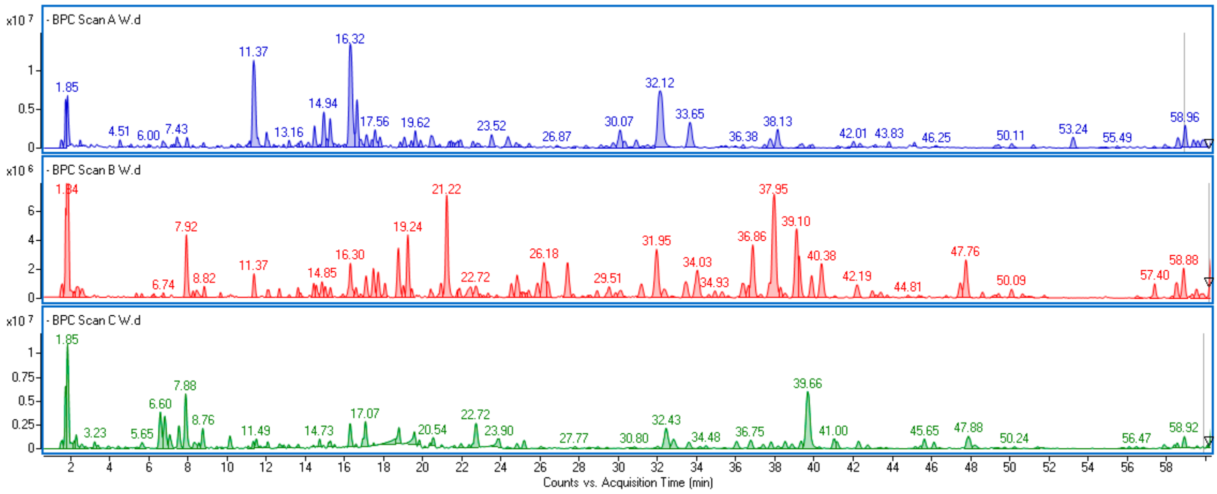

Figure S1. Base peak chromatogram of leaf extracts from *P. armeniaca* (blue line), *P. cerasus* (red line), and *M. domestica* (green line).

Table S1. Quantitative analysis of *P. armeniaca* leaf extract performed using UHPLC/DAD/ESI-MS. The values represent means ± standard deviation (SD) of triplicate.

R_T (min.)	m/z-H (Fragments)	Δ ppm	Formula	Compound	PAWE (µg/mL)	PAEE (µg/mL)
1.82	191.05699	4.57	$C_7H_{12}O_6$	Quinic acid	+	+
8.80	153.01952	1.22	$C_7H_6O_4$	Protocatechuic acid*	3.39 ± 0.1	2.01 ± 0.11
11.40	353.08788 (179, 191)	0.21	$C_{16}H_{18}O_9$	Neochlorogenic acid*	280.3 ± 6.5	445.9 ± 11.5
12.61	325.09295	0.18	$C_{15}H_{18}O_8$	p-Coumaric acid glucoside	+	+
13.78	337.09291 (163, 191, 173)	0.06	$C_{16}H_{18}O_8$	3-p-Coumaroylquinic acid	4.59 ± 0.30	7.89 ± 0.51
14.46	337.09266 (163, 191, 173)	-0.68	$C_{16}H_{18}O_8$	3-p-Coumaroylquinic acid	39.97 ± 3.83	38.97 ± 1.41
16.31	353.08909 (191, 179)	3.63	$C_{16}H_{18}O_9$	Chlorogenic acid*	445.5 ± 22.1	718.5 ± 22.1
16.36	353.08895 (191, 179)	3.23	$C_{16}H_{18}O_9$	Cryptochlorogenic acid*	86.49 ± 1.95	137.5 ± 9.92
16.95	179.03501	0.15	$C_9H_8O_4$	Caffeic acid*	8.17 ± 011	5.71 ± 0.42
19.88	337.09311 (191,173)	0.65	$C_{16}H_{18}O_8$	4-p-Coumaroylquinic acid	17.03 ± 1.11	19.34 ± 1.64
20.43	337.09367 (191,173)	2.30	$C_{16}H_{18}O_8$	5-p-Coumaroylquinic acid	27.12 ± 0.81	31.20 ± 1.97
21.71	367.10382	0.99	$C_{17}H_{20}O_9$	Feruloylquinic acid	5.61 ± 0.15	12.23 ± 0.30
22.58	367.10398	1.42	$C_{17}H_{20}O_9$	Feruloylquinic acid	5.48 ± 0.62	6.31 ± 0.39
22.79	337.09394 (191, 173)	3.10	$C_{16}H_{18}O_8$	5-p-Coumaroylquinic acid	9.10 ± 0.35	8.25 ± 0.23
26.24	193.05097	1.74	$C_{10}H_{10}O_4$	Ferulic Acid*	3.24 ± 0.17	4.34 ± 0.12
30.92	609.14676	1.07	$C_{27}H_{30}O_{16}$	Quercetin 7-O-rutinoside*	4.91 ± 0.32	9.48 ± 0.19
32.04	609.14682	1.17	$C_{27}H_{30}O_{16}$	Quercetin 3-O-rutinoside*	136.20 ± 5.49	632.40 ± 14.41
33.65	463.08934 (301, 300)	2.46	$C_{21}H_{20}O_{12}$	Quercetin 3-O-glucoside*	9.61 ± 0.53	35.04 ± 1.45
37.75	505.09832 (463, 300)	-0.88	$C_{23}H_{22}O_{13}$	Quercetin acetyl hexoside	2.43 ± 0.12	7.53 ± 0.55
38.14	593.15168	0.82	$C_{27}H_{30}O_{15}$	Kaempferol-3-O-rutinoside*	10.72 ± 0.48	36.66 ± 1.53
39.40	447.09401	1.62	$C_{21}H_{20}O_{11}$	Kaempferol-3-O-glucoside*	3.99 ± 0.17	7.32 ± 0.51
39.78	447.09433 (300)	2.33	$C_{21}H_{20}O_{11}$	Quercitrin*	+	+
41.32	505.09694 (300)	-3.6	$C_{23}H_{22}O_{13}$	Quercetin acetyl hexoside	+	+
42.34	435.09505	4.05	$C_{20}H_{20}O_{11}$	unknown	+	+

*- identification was confirmed using a standard.

Table S2. Quantitative analysis of *P. cerasus* leaf extract performed using UHPLC/DAD/ESI-MS. The values represent means ± standard deviation (SD) of triplicate.

R_T (min.)	m/z-H (Fragments)	Δ ppm	Formula	Compound	PCWE (µg/mL)	PCEE (µg/mL)
1.83	191.05674	3.27	$C_7H_{12}O_6$	Quinic acid	+	+
6.74	299.07611 (137)	-3.77	$C_{13}H_{16}O_8$	Hydroxybenzoic acid glucoside	+	+
7.90	315.07356	4.44	$C_{13}H_{16}O_9$	Dihydroxybenzoic acid hexoside	+	+
8.70	153.01967	2.19	$C_7H_6O_4$	Protocatechuic acid*	4.43 ± 0.29	1.46±0.51
11.35	353.08797 (179, 191)	0.46	$C_{16}H_{18}O_9$	Neochlorogenic acid*	12.05 ± 0.65	27.24±1.28
12.58	325.09311	0.67	$C_{15}H_{18}O_8$	p-Coumaric acid glucoside	+	+
13.63	337.09321 (163, 191, 173)	0.94	$C_{16}H_{18}O_8$	3-p-Coumaroylquinic acid	4.53 ± 0.15	8.28±0.45
14.39	337.09354 (163, 191, 173)	1.92	$C_{16}H_{18}O_8$	3-p-Coumaroylquinic acid	16.13 ± 065	33.57±1.02
14.98	325.09301	0.36	$C_{15}H_{18}O_8$	p-Coumaric acid glucoside	2.22 ± 0.12	38.49±1.16
15.63	289.07218 (221, 245)	1.44	$C_{15}H_{14}O_6$	Catechin*	+	+
16.26	353.08897 (191, 179)	3.29	$C_{16}H_{18}O_9$	Chlorogenic acid*	12.38 ± 0.51	115.0±7.21
16.58	353.08901 (191, 179)	3.40	$C_{16}H_{18}O_9$	Cryptochlorogenic acid*	5.41 ± 0.12	12.02±0.67
19.24	655.22198 (327)	-3.62	$C_{30}H_{40}O_{16}$	unknown	+	+
19.53	289.07255 (221, 245)	2.72	$C_{15}H_{14}O_6$	Epicatechin*	+	+
19.79	337.09357 (191, 173)	2.01	$C_{16}H_{18}O_8$	4-p-Coumaroylquinic acid	1.59 ± 0.05	1.80 ± 2.41
20.34	337.09349 (191, 173)	1.77	$C_{16}H_{18}O_8$	5-p-Coumaroylquinic acid	3.87 ± 0.21	24.04 ± 1.02
21.24	325.09322	1.01	$C_{15}H_{18}O_8$	p-Coumaric acid glucoside	57.65 ± 2.25	58.24 ± 2.02
22.46	367.10401	1.51	$C_{17}H_{20}O_9$	Feruloylquinic acid	1.94 ± 0.08	3.83 ± 0.32
22.77	337.09399 (191, 173)	3.25	$C_{16}H_{18}O_8$	5-p-Coumaroylquinic acid	11.73 ± 1.00	4.23 ± 0.31
24.56	771.20248 (463)	4.59	$C_{33}H_{40}O_{21}$	Quercetin deriv.	2.10 ± 0.11	15.80 ± 0.68
24.86	755.20181	-2.92	$C_{33}H_{40}O_{20}$	Quercetin deriv.	4.31 ± 0.25	27.39 ± 1.54
27.47	785.2166	2.64	$C_{34}H_{42}O_{21}$	Isorhamnetin deriv.	18.43 ± 0.87	34.06 ± 1.58
29.57	433.11521 (271)	2.74	$C_{21}H_{22}O_{10}$	Naringenin-7-O-glucoside*	2.95 ± 0.11	24.32 ± 0.85
30.84	609.14691	1.31	$C_{27}H_{30}O_{16}$	Quercetin 7-O-rutinoside*	+	+
32.02	609.14701 (300)	1.48	$C_{27}H_{30}O_{16}$	Quercetin 3-O-rutinoside*	17.33 ± 0.43	73.88 ± 2.51
33.52	463.08951 (300)	2.82	$C_{21}H_{20}O_{12}$	Quercetin 3-O-glucoside*	2.05 ± 0.11	15.49 ± 0.78
35.02	593.15201	1.37	$C_{27}H_{30}O_{15}$	Kaempferol deriv.	+	+
36.17	431.09901 (269)	1.48	$C_{21}H_{20}O_{10}$	Apigenin hexoside	+	+
37.35	515.12045 (353)	1.84	$C_{25}H_{24}O_{12}$	Dicaffeoylquinic acid	+	5.13 ± 0.25
37.98	593.15203	1.41	$C_{27}H_{30}O_{15}$	Kaempferol-3-O-rutinoside*	59.48 ± 2.05	250.9 ± 20.14
39.03	515.12069 (353)	2.31	$C_{25}H_{24}O_{12}$	3,5-Dicaffeoylquinic acid*	+	73.01 ± 3.24
39.17	623.16076	-1.60	$C_{28}H_{32}O_{16}$	Isorhamnetin deriv.	50.9 ± 2.23	53.6 ± 2.96
39.41	447.09431	2.29	$C_{21}H_{20}O_{11}$	Kaempferol-3-O-glucoside*	+	+
40.45	477.10601	4.52	$C_{22}H_{22}O_{12}$	Isorhamnetin hexoside	4.46 ± 0.24	8.69 ± 0.41
42.88	515.12021 (353)	1.38	$C_{25}H_{24}O_{12}$	4,5-Dicaffeoylquinic acid*	+	20.20 ± 1.08

*- identification was confirmed using a standard.

Table S3. Quantitative analysis of *M. domestica* leaf extract performed using UHPLC/DAD/ESI-MS. The values represent means ± standard deviation (SD) of triplicate.

R_T (min.)	m/z-H (Fragments)	Δ ppm	Formula	Compound	MDWE (µg/mL)	MDEE (µg/mL)
1.82	191.05656	2.33	$C_7H_{12}O_6$	Quinic acid	+	+

7.94	315.07301	2.70	$C_{13}H_{16}O_9$	Dihydroxybenzoic acid hexoside	+	+
8.75	153.02001	4.40	$C_7H_6O_4$	Protocatechuic acid*	13.14 ± 0.10	2.39 ± 0.09
9.50	299.07702 (137)	-0.74	$C_{13}H_{16}O_8$	Hydroxybenzoic acid glucoside	+	+
11.20	353.08811 (179, 191)	0.86	$C_{16}H_{18}O_9$	Neochlorogenic acid*	3.39 ± 0.11	3.05 ± 0.12
15.20	325.09318	0.89	$C_{15}H_{18}O_8$	p-Coumaric acid glucoside	+	+
16.30	353.08871 (191, 179)	2.55	$C_{16}H_{18}O_9$	Chlorogenic acid*	14.86 ± 0.57	29.96 ± 1.38
16.58	353.08912 (191, 179)	3.71	$C_{16}H_{18}O_9$	Cryptochlorogenic acid*	3.13 ± 0.12	3.27 ± 0.21
17.07	179.03528	0.65	$C_9H_8O_4$	Caffeic acid*	11.57 ± 0.91	3.47 ± 0.18
19.85	337.09385 (191, 173)	2.84	$C_{16}H_{18}O_8$	4-p-Coumaroylquinic acid	8.68 ± 0.32	9.23 ± 0.78
20.37	337.09355 (191, 173)	1.95	$C_{16}H_{18}O_8$	5-p-Coumaroylquinic acid	5.76 ± 0.37	14.22 ± 0.51
21.41	449.10951	1.28	$C_{21}H_{22}O_{11}$	Eriodictyol hexoside	+	62.41 ± 2.71
22.79	337.09371 (191, 173)	2.42	$C_{16}H_{18}O_8$	5-p-Coumaroylquinic acid	5.25 ± 0.31	6.51 ± 0.41
22.96	449.10967	1.63	$C_{21}H_{22}O_{11}$	Eriodictyol hexoside	+	69.19 ± 3.51
27.58	495.11521	1.60	$C_{22}H_{24}O_{13}$	Catechin deriv.	+	+
32.11	609.14725	1.87	$C_{27}H_{30}O_{16}$	Quercetin 3-O-rutinoside*	+	8.91 ± 0.62
32.41	463.08963 (301, 300)	3.08	$C_{21}H_{20}O_{12}$	Quercetin 3-O-galactoside*	5.33 ± 0.20	47.17 ± 1.82
33.56	463.08911 (301, 300)	1.96	$C_{21}H_{20}O_{12}$	Quercetin 3-O-glucoside*	1.20 ± 0.08	37.95 ± 1.21
35.49	451.12487 (289)	0.63	$C_{21}H_{24}O_{11}$	(epi)catechin hexoside	+	1286.1 ± 52.5
36.04	433.07798	0.79	$C_{20}H_{18}O_{11}$	Quercetin pentoside	1.26 ± 0.09	7.74 ± 0.61
38.14	433.07821	1.32	$C_{20}H_{18}O_{11}$	Quercetin-3-O-arabinoside*	0.61 ± 0.04	27.45 ± 1.16
38.86	433.07837	1.69	$C_{20}H_{18}O_{11}$	Quercetin pentoside	0.73 ± 0.06	4.73 ± 0.28
39.32	447.09402	1.64	$C_{21}H_{20}O_{11}$	Kaempferol-3-O-glucoside*	20.64 ± 1.62	79.89 ± 3.21
39.67	447.09431	2.29	$C_{21}H_{20}O_{11}$	Quercetin 3-rhamnoside*	2.91 ± 0.09	10.45 ± 1.00
42.07	435.13005 (273)	0.87	$C_{21}H_{24}O_{10}$	Phloridzin*	1.56 ± 0.01	2584.2 ± 54.9
45.65	431.09845	0.18	$C_{21}H_{20}O_{10}$	Kaempferol deriv	0.54 ± 0.04	11.69 ± 1.01
47.85	287.05657 (151)	1.59	$C_{15}H_{12}O_6$	Eriodictyol*	20.39 ± 1.01	120.9 ± 3.24
57.95	273.07699	0.52	$C_{15}H_{14}O_5$	Phloretin*	+	86.65 ± 3.21
61.24	581.16501 (273)	-2.47	$C_{30}H_{30}O_{12}$	Phloretin coumaroyl-5-O-glucoside	+	87.08 ± 3.54
61.88	611.17787 (329, 273)	1.40	$C_{31}H_{32}O_{13}$	Phloretin feruloyl-5-O-glucoside	+	+
62.53	597.16144 (273)	0.13	$C_{30}H_{30}O_{13}$	Phloretin caffeoyl-6-O-glucoside	+	+

*- identification was confirmed using a standard.

Table S4. ABTS and DPPH radical scavenging IC_{50} values for *M. domestica*, *P. armeniaca* and *P. cerasus* leaf extracts. The values were determined for extracts from *M. domestica* leaves (water (MDWE), water-ethanol (MDEE)), extracts from *P. armeniaca* leaves (water (PAWE), water-ethanol (PAEE)) and extracts from *P. cerasus* leaves (water (PCWE), water-ethanol (PCEE)). Values are means ± standard deviation (SD) of triplicates.

	**ABTS [µg/mL] ± SD**	**DPPH [µg/mL] ± SD**
MDWE	37.5 ± 0.08	38.5 ± 0.09
MDEE	14.5 ± 0.03	32.5 ± 0.08
PAWE	47.5 ± 0.11	90.0 ± 0.22
PAEE	21.0 ± 0.05	35.5 ± 0.09
PCWE	78.0 ± 0.19	205.0 ± 0.49
PCEE	33.5 ± 0.08	72.5 ± 0.05
